# Comparative analysis of the uptake of the H^+^/OC antiporter substrate oxycodone across and into brain endothelial and parenchymal cells with in vitro–in vivo extrapolation

**DOI:** 10.1186/s12987-025-00726-w

**Published:** 2025-10-30

**Authors:** Frida Bällgren, Nana Svane, Aghavni Ginosyan, Alberte Bay Villekjaer Pedersen, Shannuo Li, Jessica Mahajan, Morten Schallburg Nielsen, Birger Brodin, Irena Loryan

**Affiliations:** 1 Translational Pharmacokinetics/Pharmacodynamics Group (tPKPD), Department of Pharmacy, Uppsala University, Husargatan 3, Uppsala, 752 37 Sweden; 2 Present Address: Department of Pharmacy, Science for Life Laboratory Drug Discovery and Development (SciLifeLab DDD), Uppsala University, Uppsala, Sweden; 3 CNS Drug Delivery and Barrier Modelling Group (CNSBM), Department of Pharmacy, University of Copenhagen, Copenhagen, Denmark; 4 Present Address: Department of Molecular Pharmaceutics, College of Pharmacy, University of Utah, Salt Lake City, UT 84112 USA; 5 Present Address: School of Applied Sciences, Abertay University, Bell Street, Dundee, Scotland, DD1 1HG UK; 6 Department of Biomedicine, Aarhus University, Aarhus, Denmark

**Keywords:** Putative proton-coupled organic cation (H^+^/OC) antiporter, Oxycodone, Pyrilamine, hCMEC/D3, Primary brain endothelial cells, Brain slice, K_p,Uu,Cell_, Rate, Extent, Pig, Rat, Mouse

## Abstract

**Background:**

In vitro evaluation of substances utilizing the putative proton‑coupled organic cation (H^+^/OC) antiporter for active uptake across the blood-brain barrier (BBB) and brain cell membranes requires a thorough understanding of cellular pharmacokinetics supported by reliable translational readouts. This study assessed the rate and extent of uptake of the antiporter substrate oxycodone in brain endothelial and parenchymal cells at clinically relevant concentrations, exploring the suitability of various cell models for investigating active drug transport.

**Methods:**

Transcellular transport studies were performed using primary brain endothelial cells (BECs) from pig, rat, and mouse, alongside uptake assays in immortalized human cerebral microvascular endothelial cells (hCMEC/D3) and rat brain slices. Drug uptake was estimated by combining transport data with non-specific binding data via equilibrium dialysis. The effect of interleukin-6 (IL-6) on oxycodone uptake was tested in hCMEC/D3 cells. The unbound intracellular-to-extracellular concentration ratio (K_p,uu,cell_) and efflux ratio were used to compare the extent of net uptake across models and evaluate the presence of active uptake.

**Results:**

Based on cellular pharmacokinetic parameters, both primary BECs and hCMEC/D3 demonstrated active uptake of oxycodone. Mean permeability across primary BECs ranged between 0.9 × 10⁻⁵ (pig) and 1.8 × 10⁻⁵ (rat) cm/s. Transport extent, reflected by 1/Efflux Ratio values of 1.5 (pig) and 2.4 (rat), aligned with in vivo unbound brain-to-plasma concentration ratios, K_p,uu,brain_, indicating predominant active uptake. In hCMEC/D3 cells, the uptake rate was time- and concentration-dependent within 60 min and 10–5000 nM concentration range, while the uptake extent, assessed by K_p,uu,cell_, was 1.7 and independent of both. IL-6 increased the extent of uptake to 148% of control, without affecting the rate. The extent of uptake into parenchymal cells was also concentration-independent, with K_p,uu,cell_ values around 1.8, similar to endothelial cells.

**Conclusions:**

These findings provide insight into oxycodone distribution across the BBB and into brain parenchymal cells, particularly emphasizing the contribution of probable active transport mechanisms at multiple barriers. The results highlight the importance of assessing both the rate and extent of transport and support the utility of K_p,uu,cell_ as a key metric for comparing drug transport across models. Both primary BECs and hCMEC/D3 cells are reliable tools for assessing substrate uptake in drug development, particularly for H^+^/OC antiporter substrates, and hold translational potential for mechanistic studies.

**Graphical Abstract:**

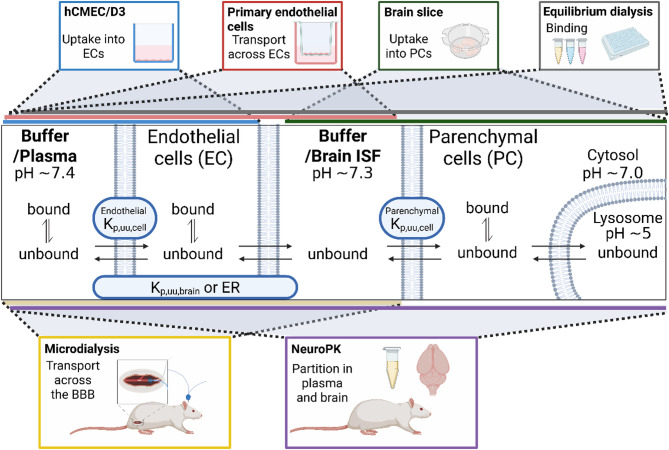

**Supplementary Information:**

The online version contains supplementary material available at 10.1186/s12987-025-00726-w.

## Introduction

Targeting active uptake transporters at the blood-brain barrier (BBB) is a strategy to increase the likelihood of reaching a sufficient CNS exposure to obtain a therapeutic effect while minimizing peripheral adverse events. A promising active uptake system at the BBB is the putative proton- coupled organic cation (H^+^/OC) antiporter [1, 2], although the mechanism of antiporter-mediated uptake is not fully understood. Traditionally, functional characterization of the H^+^/OC antiporter is based on a set of experiments confirming saturable transport, temperature dependence, cis-inhibition, trans-stimulation, pH dependence, sodium independence [1, 3–7]. Based on these characteristics, several well-known drugs, including oxycodone [3] and pyrilamine [8, 9], have been identified as substrates of this antiporter. Pharmacokinetic studies offer an alternative approach to characterize active uptake phenomena. Numerous in vivo studies have reported unbound brain-to-plasma concentration ratios (K_p,uu,brain_) above unity for these substrates, e.g., 2.5 (pigs), 3-4.4 (rats), and 2 (mice) for oxycodone, suggesting an active uptake across the BBB, i.e., a net influx from the systemic circulation to brain interstitial fluid (ISF) [10–13]. Variability in H^+^/OC antiporter substrate uptake across in vitro BBB models of different origins has been sparsely investigated. As a result, a critical knowledge gap remains regarding the presence and functional relevance of the H⁺/OC antiporter system in commonly used in vitro BBB models, and how well these models replicate in vivo conditions.

**Fig. 1:**
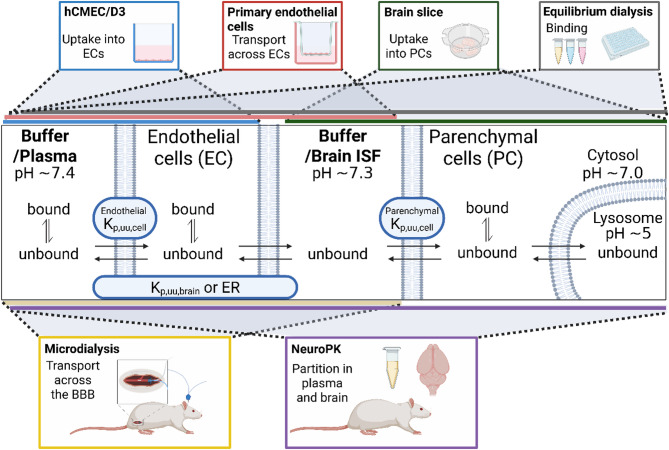
Schematic representation of in vitro and in vivo methods for investigation of the key processes of brain drug disposition employed in the present study. In vitro methods included are hCMEC/D3 (blue box), primary endothelial cells (red box), brain slice (green box), and equilibrium dialysis (grey box). The in vivo method included microdialysis (yellow box). Neuropharmacokinetic (neuroPK) brain distributional study (purple box) is included as the most commonly used in vivo approach. The colored bars indicate the compartments that each method could characterize. The hCMEC/D3, primary endothelial cells, and microdialysis methods investigate uptake into and/or transport across brain endothelial cells (ECs), while the brain slice method focuses on uptake into brain parenchymal cells (PCs). Plasma (in vivo), mimicked by buffer (in vitro), interacts with the apical side of ECs. The blood-brain barrier, consisting of multiple cell types in vivo, is modeled by only endothelial cells in vitro. The brain interstitial fluid (ISF; in vivo), mimicked by buffer (in vitro), faces the basolateral side of ECs, and PC membranes. Within these compartments, the drug may be bound or unbound, where unbound is available for transport across membranes by passive or active transport in both directions (indicated by arrows). The proton-coupled organic cation (H^+^/OC) antiporter is functionally confirmed at the apical EC membrane, while at other membranes, it remains poorly understood (marked by question marks). For organic cations, passive transport of unionized moieties follows pH partitioning (NB: pH values for compartments are provided where known). The uptake of drugs into ECs can be quantified using the unbound EC-to-buffer concentration ratio (endothelial K_p,uu,cell_). Following uptake, the drug can be released at the basolateral endothelial membrane into the brain ISF or buffer. The extent of transport across endothelial cells is determined by the unbound brain ISF-to-plasma concentration ratio (K_p,uu,brain_), or calculated from the inverted efflux ratio (ER), providing an estimate of the in vitro K_p,uu,brain_. For PCs, the unbound drug can cross the apical membrane and distribute into the cytosol. Parenchymal K_p,uu,cell_ describes the unbound PC intracellular-to-buffer (brain ISF) concentration ratio. Drugs may be distributed into subcellular compartments like lysosomes, where the unbound PC lysosome-to-cytosol concentration ratio (K_p,uu,lyso_) can be estimated [74]

To enable meaningful comparisons between cell types and to perform appropriate in vitro – in vivo (IVIV) extrapolation, cellular pharmacokinetic readouts must be of the same kind. CNS drug distribution from blood involves several processes, including transport of unbound drug into brain endothelial cells, from endothelial cells to the brain ISF, and into brain parenchymal cells and subcellular organelles (Fig. 1). In parallel, in all physiological compartments, non-specific and specific binding may occur. It is important to bear in mind that readouts characterizing various aspects of drug distributional processes, such as rate vs. extent of uptake, cannot be directly compared. For example, as discussed by Hammarlund-Udenaes et al. [14], high drug permeability (rate) does not necessarily translate to a high extent of brain delivery. Yet, traditionally, in vitro studies often focus on the rate of transport, whereas in vivo studies often focus on the clinically more relevant extent of transport, highlighting the need to improve the practice of investigating the extent of transport in in vitro models.

To exert its CNS effect in vivo, a drug must reach a certain exposure level at brain ISF and/or intracellular target sites, making it crucial to quantify the extent of unbound drug distribution to these compartments (Fig. 1). While this is a complex process, several aspects of the extent of distribution can be investigated in dedicated, fit-for-purpose, in vitro studies. The extent of drug transport across the BBB in vitro is routinely assessed using the efflux ratio (ER), defined as the ratio of the drug’s permeability in the basolateral-to-apical (B-to-A) to that in the apical-to-basolateral (A-to-B) direction across an endothelial monolayer [15]. ER evaluates the potential of a drug candidate to cross the BEC monolayer and is often used in drug candidate selection, where values below two are typically considered favorable for CNS delivery [16–19] and values below unity are potentially associated with active uptake. ER inversely correlates to K_p,uu,brain_ measured in vivo, which is accepted as the gold standard for assessment of the extent of BBB transport [14, 20, 21]. However, discrepancies can arise between in vitro ER values and in vivo K_p,uu,brain_ values [22]. Feng et al. highlighted several compounds with active uptake in vivo, and still an ER above unity in vitro. Additionally, ER values for the same compound may vary across BBB models in vitro, resulting in inconsistent BBB permeability classifications [22]. This further emphasizes the need to use robust models with reliable IVIV correlations.

The extent of drug transport into the brain endothelial cell (BEC) from the blood side can be quantified in vitro using the unbound intra-to-extracellular concentration ratio, K_p,uu,cell_, with values above unity indicating predominant active uptake (see Materials and Methods). Although first presented in 2007 by Fridén et al. [23] and since widely accepted for characterization of cellular pharmacokinetics in various in vitro models, to our knowledge, the use of the K_p,uu,cell_ metric is very limited in the BBB field. In addition, as originally proposed, the drug transport from ISF into brain parenchymal cells could also be assessed in vitro using brain parenchymal K_p,uu,cell_ by employing the brain slice method [24, 25]. When comparing endothelial and brain parenchymal cellular transport processes, it is important to recognize that they form separate barriers, each requiring dedicated investigation to capture their individual contributions to CNS drug distribution. A simple BBB cell model that includes both the drug uptake and release from BECs, and therefore may be used for assessment of both the rate and extent of transport across the BBB, is primary BECs cultured in a transcellular set-up [26–28]. Yet, transport studies of antiporter substrates in transcellular set-ups are sparse. In this study, transcellular models with primary pig, rat, and mouse BECs (pBECs, rBECs, mBECs) were utilized to explore potential variabilities in uptake across the models, and their respective in vivo correlation. The hCMEC/D3 cell line is an easily grown, simple, and stable human BBB model, commonly used to investigate uptake mechanisms and interactions under various conditions, e.g., incubation at 4 °C, pH modulation [29, 30]. Still, due to limitations, including incomplete tight junction integrity [15, 31], it is merely suitable for cellular uptake rather than transcellular transport studies. Yet, it remains unclear whether such uptake studies can still be useful in predicting BBB transport in control and pathological conditions. In vivo studies have shown that inflammation can influence H^+^/OC antiporter-mediated uptake across the BBB [13, 32, 33]. With interleukin 6 (IL-6) being one of the key proinflammatory cytokines released during inflammation in vivo [33–36], in the present study, uptake into hCMEC/D3 cells was also evaluated in the presence of IL-6 to assess its impact on the uptake.

Although antiporter-mediated uptake (primarily rate) has been studied in various in vitro BBB models, including immortalized human brain capillary endothelial cells (hCMEC/D3) [4–6, 37, 38], immortalized rat brain capillary endothelial cells (TR-BBB13) [3, 39], primary bovine BECs [7, 9], primary rat BEC [32], and more complex models as the microfluidic neurovascular unit model with human induced pluripotent stem cell-derived BECs [40], there is a lack of systematic investigation of the same substrate across different models followed by IVIV extrapolation. In addition, in these models, uptake is often studied at high substrate concentrations, making it unclear whether a similar extent of uptake occurs at clinically relevant exposure levels. Using in vivo-relevant concentrations in vitro is an important step towards unifying experimental conditions.

The main aim of this study was to evaluate the extent to which in vitro uptake of H^+^/OC antiporter substrate reflects in vivo brain drug delivery, by using oxycodone as a model substrate with reported data on its CNS disposition in pigs, rats, and mice in healthy and endotoxemic conditions as well as using cellular pharmacokinetic parameters to guide conclusions. To support this, substrate uptake into and across brain endothelial cells of different origins, and into brain parenchymal cells, was evaluated in vitro. This was achieved using the human cell line hCMEC/D3, primary BECs from pigs, rats, and mice, and the brain slice method applied to rats. The impact of inflammatory signaling on drug uptake was also examined by assessing the effect of the key in vivo cytokine IL-6 on the substrate uptake into hCMEC/D3. We found that the antiporter substrate oxycodone is actively distributed into and across BBB cell models of human, pig, rat, and mouse origins, as well as into rat brain parenchymal cells. Data demonstrate that transport may vary across models of different origins. We confirm that K_p,uu,cell_ is a valuable metric for comparing drug transport across different experimental models, as it characterizes membrane transport of unbound drug and is independent of drug non-specific binding. According to our findings, these in vitro models can predict in vivo brain drug delivery and have the potential to be used as tools for screening for active drug uptake in drug development, facilitating the selection of drug candidates during development.

## Materials and methods

### Chemicals

Oxycodone hydrocloride (OxyNorm^®^, Mundipharma, Cambridge, England) was purchased from Distansapoteket Stockholm (Apoteket AB, Stockholm, Sweden). Oxycodone hydrochloride (HPLC grade reference standard, Eur. Qual D, APL, Kungens Kurva, Sweden) was purchased from Distansapoteket (Falun, Sweden). Oxycodone hydrochloride (British Pharmacopoeia reference standard), pyrilamine maleate salt, IL-6 (GF338, Human Recombinant Animal Free), Hanks′ Balanced Salt solution (HBSS), ascorbic acid, human basic fibroblast growth factor (bFGF), and hydrocortisone (H4001) were purchased from Merck (Soeborg, Denmark). Oxycodone-D3 and oxycodone-D6 (Cerriliant), phosphate-buffered saline (PBS, D8537, Sigma Life Science, USA), EBM^®^-2 medium (Lonza, Basel, Switzerland), Triton™ X-100, Erythrosine B, dextran (Leuconostoc mesenteroides; 64000–76000 kDa, D8821), collagen type I, collagen type IV from human placenta (C5533), fibronectin from human plasma (F1056, for rBEC), puromycin dihydrochloride (10 mg/mL in water, from Streptomyces alboniger, P9620 for rBEC, P8833 for pBEC), Trypsin-EDTA (T4299, for rBEC), RO (4-(3-Butoxy-4-methoxybenzyl)imidazolidin-2-one, B8279), cAMP (8-(4-Chlorophenylthio)adenosine 3′,5′-cyclic monophosphate sodium salt, C3912 for rBEC, C010-500 for pBEC), dexamethasone (D4902), HEPES (4-(2-hydroxyethyl)-1-piperazineethanesulfonic acid, 1 M, pH 7.0-7.6, sterile-filtered, H0887), penicillin/streptomycin (pen/strep, 10 000 U/mL /10 mg/mL in 0.9% NaCl, P0781, Gibco, Life Technologies, USA, for rBEC), Dulbecco’s Modified Eagle’s Medium (DMEM) – high glucose (4500 mg/L glucose, L-glutamine, and sodium bicarbonate, without sodium pyruvate, liquid, sterile-filtered, D5796), powdered Dulbecco’s Modified Eagle’s Medium – high glucose (4500 mg/L glucose and L-glutamine, without sodium bicarbonate, D5648), N-[Tris(hydroxymethyl)methyl]-2-aminoethanesulfonic acid (TES free acid, 93359), EDTA (E6511 Sigma, for pBEC), heparin (H3149-50KU, Sigma), collagen IV (C5533, Sigma), fetal bovine serum (FBS, F9665, Sigma, for pBEC), sodium chloride, potassium chloride, magnesium chloride, calcium dichloride, ascorbic acid, and potassium dihydrogen phosphate, isopentane were purchased from Sigma-Aldrich (Stockholm, Sweden). Collagenase III, Trypsin TRL, and DNAse I were purchased from Worthington Biochemical Corporation (New Jersey, US). MEM non-essential amino acids mixture (11140050), chemically defined lipid concentrates, pen/strep (10 000 U/mL /10 mg/mL in 0.9% NaCl, Gibco, Life Technologies, USA, 15140-122, used for pBEC culturing), and Trypsin (2.5%, 10x, Gibco, Life Technologies, USA, 15090-046, used for pBEC culturing) were purchased from Thermo Fisher Scientific (Waltham, Massachusetts, USA). Standard fetal bovine serum (FBS, SH30088.03, for rBEC and hCMEC/D3) was purchased from PAA laboratories (GE Healthcare Life Sciences, Hyclone Laboratories, USA. Ultima Gold 241™ and ^3^H-pyrilamine were purchased from Perkin Elmer (MA, USA). Dulbecco’s Modified Eagle’s Medium/Nutrient Mixture F-12 (DMEM/F12, 392–0412, for pBEC) was purchased from VWR International (Soeborg, Denmark). Sterile Plasma Derived Bovine Serum (PDS) was purchased from First Link Ltd. (Birmingham, UK). Bovine Fibronectin Protein (CF, 1030-FN, for pBEC) was purchased from R&D Systems, Inc. (Abingdon, UK).

### Brain endothelial cell transport and uptake

To evaluate oxycodone and ^3^H-pyrilamine transport in BBB cell models, primary BEC models and hCMEC/D3 were used. Drug transport across and into primary BECs was assessed through transcellular transport studies with monocultured pBEC and rBEC, and a pilot study in mBEC. To evaluate the impact of IL-6 on drug uptake, uptake studies were conducted in hCMEC/D3 cells with and without the presence of IL-6.

#### Isolation and culturing of primary brain endothelial cells

Isolation of primary rBECs and mBECs was performed at the University of Copenhagen, Denmark. Four-week-old Sprague-Dawley rats (*N* = 12, *N* = 4 per batch, three batches, Envigo, Horst, Netherlands) of both sexes, and male C57BL/6JRccHsd mice (*N* = 8, *N* = 8 per batch, one batch, Envigo, Horst, Netherlands) were used to obtain BECs. The animals were group-housed by species and sex under controlled conditions (20–22 °C and 45–65% humidity) with a 12-hour light/dark cycle and free access to food and water. The animals were acclimatized for two weeks before brain isolation. The isolation of rBEC and mBECs was performed as described previously [41]. The mice were sacrificed by cervical dislocation while the rats were anesthetized in a CO_2_ chamber (70% for up to 2 min), thereafter, the animals were dipped in ethanol and decapitated. The brain was isolated, meninges were removed, and the tissue was homogenized in ice-cold DMEM supplemented with pen/strep (100 U/mL /0.1 mg/mL) using a Dounce tissue grinder (40 mL volume, Sigma – D9188). The homogenate was diluted 1:1 (v: v) in 32% (w: v) dextran in DMEM, and centrifuged at 2400×g for 20 min at 4 °C. Myelin and tissue debris were discarded, and the capillary-containing fraction was re-suspended in 10% FBS in DMEM, followed by centrifugation at 800×g for 10 min at 4 °C. Capillaries in the supernatant were filtered through a nylon filter that was pre-wet with 10% FBS in DMEM (41 μm nylon mesh; diameter: 47 mm, Millipore – NY4104700) using a Swinnex Filter Holder (47 mm, Millipore, Sigma SX0004700), and washed off with 10% FBS in DMEM. The capillary suspension was then centrifuged at 500×g for 5 min at room temperature. The pellet was digested with a mix of trypsin (105 U/mL), DNAse I (170 U/mL) and collagenase type III (200 U/mL), in 10% FBS in DMEM for 40–60 min on water bath at 37 °C, thereafter, suspended in 10% FBS in DMEM and centrifuged at 1000×g for 3 min at room temperature. The capillary suspension was then transferred to collagen and fibronectin-coated T25 flasks for incubation at normal incubation conditions of 37 °C with 5% CO_2_ (430639, 25 cm², rectangular, canted neck, cell culture flask with 2 μm Vent Cap, polystyrene, sterile, nonpyrogenic, Corning^®^, Steuben County, USA). Capillary fragments were settled for 2–3 h before the media was replaced with DMEM containing 10% FBS and 4 µg/mL puromycin. On day 3, the media was changed to 10% FBS in DMEM. On day 5, the BECs were detached from the flask with trypsin-EDTA, centrifuged in 10% FBS in DMEM at 1000× g for 10 min at room temperature, re-suspended in 10% FBS in DMEM, and seeded on the apical side of the permeable cell culture supports (42 000 cells/support; 0.336 cm² ThinCert™, Greiner Bio-One GmbH, Germany). On day 8, the media was changed to differentiation media (50 mM TES free acid in DMEM supplemented with, 10% FBS, pen/strep 100 U/mL/ 0.1 mg/mL, 1% non-essential amino acid mixture, 4.9 µg/mL cAMP, 18 µg/mL RO, 0.20 µg/mL dexamethasone, pH 7.4). During the culture period, the cells were maintained at normal incubation conditions. By day 10, the rBEC and mBEC models were ready for use, which was confirmed by transendothelial electrical resistance (TEER) measurements performed at room temperature before the start of the experiment (Endohm 12, cup electrode chamber connected to an EVOM voltmeter). To obtain the absolute TEER values of the cell layer, the measured value in each well was subtracted by a control value obtained in across a blank cell culture support without cells (204–287 Ω). TEER was thereafter normalized by the surface area of the cell culture support.

To obtain pBECs, brains were obtained from 5-6-month-old domestic pigs. The brains were collected immediately after slaughter and transported to the Aarhus University on ice. Isolation of primary pBECs was performed at the University of Aarhus, Denmark, in a similar way as described for rBECs and mBECs, according to previously published protocol [42]. Briefly, meninges were removed from the brains, and gray matter was isolated, transferred to ice-cold DMEM/F12 (1:1) and homogenized using a grinder. To isolate capillaries, the homogenate was filtered through 140 μm filter-mesh, and capillary-containing filters were placed in Petri dishes. The capillaries were enzymatically digested using trypsin-EDTA (0.025%/0.1 mM), DNase (170 U/mL), and collagenase type II (200 U/mL) in DMEM/F12 for 60 min at 37 °C with orbital shaking at 180 rpm. The enzymatic reaction was halted by adding DMEM/F12 supplemented with 10% FBS and 1% pen/strep, the cell suspension was transferred to new tubes and centrifuged at 250×g, 4 °C, for 5 min. The pellet was resuspended in DMEM/F12 (10% FBS, 1% pen/strep). Thereafter, the centrifugation process was repeated. After resuspension of the pellet, the cell suspension was incubated on ice for 5 min, the supernatant was carefully transferred to new tubes, and centrifuged again. Finally, the pellet was resuspended in freezing media consisting of DMEM/F12 supplemented with 10% FBS, 1% pen/strep, and 10% DMSO, and aliquoted in cryotubes stored at -80 °C overnight. The next day, the cryotubes were stored in liquid nitrogen for storage until use.

On day 1, T75 flasks were pre-coated with a collagen IV/fibronectin solution (75 µg/mL/50 µg/mL) for initial culture, and incubated for 2 h at normal incubation conditions. After thawing, the cell suspension was plated and maintained in DMEM/F12 supplemented with 10% PDS, 1% pen/strep, and 4 µg/mL puromycin. On day 2, fresh DMEM/F12 (10% PDS, 1% pen/strep, 15 U/mL heparin, 4 µg/mL puromycin) was added, and cells were cultured until reaching 60–95% confluency. On day 5, cell culture supports (0.336 cm², ThinCert™) were coated with collagen IV/fibronectin (250 µg/mL/250 µg/mL). Cells were detached from the flask using trypsin-EDTA, resuspended in DMEM/F12 (10% PDS, 1% pen/strep, 15 U/mL heparin), and seeded on the apical side of the permeable cell culture supports (36 000 cells/support). On day 8, growth media (DMEM/F12, 1% pen/strep, 15 U/mL heparin, 550 nM hydrocortisone, 17.5 µM RO, 250 µM cAMP) was added to the basolateral compartment, while growth media supplemented with 10% PDS was introduced to the apical compartment. On day 9, TEER measurements were conducted as described above, to confirm barrier integrity and that the pBEC model was ready for use.

#### Oxycodone and ^3^H-pyrilamine transport across primary brain endothelial cells

To examine oxycodone transport across primary BECs, studies were performed using rBECs and pBECs, and a pilot study using mBECs. The functionality of the H^+^/OC antiporter was probed in the rBEC model using tracer amounts of the prototypical substrate ^3^H-pyrilamine. The BEC models were used as models of the BBB, and transport experiments across the BECs, cultured on permeable supports, were conducted in differentiation media-containing (rBEC, mBEC) or growth media-containing (pBEC) transcellular set-ups at 37 °C under normal ATP-replete conditions. The transport experiments were initiated by spiking the donor compartment (either A or B) with 200 nM oxycodone or ^3^H-pyrilamine, followed by incubation at 90 rpm (orbital shaking, Heidolph Unimax 2010, Heidolph Instruments, Schwabach, Germany) for 120 min. An incubation oxycodone concentration of 200 nM was chosen as it is in vivo-relevant, with a documented mean maximal plasma concentration in humans at 34–38 ng/mL ≈ 120 nM [43, 44]. The donor compartment was sampled at 2 and 120 min to obtain initial and terminal concentrations. Samples were collected from the receiver compartment at 20, 40, 60, 80, 100, and 120 min. After the experiment, the TEER was measured again, cells were washed with ice-cold washing buffer (10% HBSS in Milli-Q water supplemented with 0.375% sodium bicarbonate), cell culture filters were removed and cells were lysed by incubation of the filters in lysis buffer (0.1% TritonX100 in Milli-Q water) for 20–35 min. Oxycodone samples were stored at 6 °C during the experiment, thereafter, at -80 °C pending bioanalysis. ^3^H-pyrilamine samples were transferred to scintillation vials and immediately analyzed (see Bioanalysis of ^3^H-pyrilamine).

To obtain the amount of drug in each compartment of the system (donor, cells, receiver), the measured sample amounts were corrected using the sample volume and the compartment volume (Eq. 1). The estimation of the amount of a drug in the cells requires a cell volume estimation, which were based on the multiplication of a theoretical height of a cell and the surface area of the cell monolayer. A theoretical cell height of 0.55 μm was used for all BECs, which was based on the mean of previously reported values ranging between 0.2 and 0.9 μm, as determined from transmission electron microscope images of rBECs [45]. Notably, the cell height is not homogenous, i.e., estimates ranging from 1.5 to 2 μm have been documented in the perinuclear region, with smaller measurements observed in plasmalemmal processes [20]. Additionally, the cell size of primary BEC has been reported to vary between cells of different origins (pig, rat, and mouse) [46].

In the transcellular set-up, the receiver amount (A_receiver_, nmol) was calculated as follows:

1 $$\:{A}_{receiver}=\raisebox{1ex}{${A}_{receiver,sample}\:\times\:{V}_{receiver}$}\!\left/\:\!\raisebox{-1ex}{${V}_{receiver,sample}$}\right.$$

Where A is the drug amount in the receiver and the sample collected from the receiver, and V is the volume of the receiver (in liters, L) and the sample collected from the receiver. At each time point, the remaining drug amount in the receiver (A_remaining, receiver_, nmol) after withdrawal of a sample at each time point (t) was calculated as:

2 $$\:{A}_{remaining,receiver}\left(t\right)={A}_{receiver}\left(t\right)-{A}_{receiver,sample}\left(t\right)$$

The accumulated amount (A_accumulated_, nmol) of oxycodone and ^3^H-pyrilamine transported across BECs at each time point (t) was calculated as the sum of the receiver amount (A_receiver_, nmol) at that time point and all the amounts previously sampled from the receiver (A_receiver, sample_, nmol):

3 $$A_{accumulated,\,receiver}(t)=A_{receiver}(t)+\sum ^{t-1}_{n=1}A_{receiver,\,sample,\,n}$$

To assess the rate of transport across BECs, the drug was assumed to be fully unbound in the buffer, and A_accumulated_ was considered unbound. Measuring the unbound drug is crucial to isolate the transport across the BECs without interference from binding. Hence, the violation of the assumption regarding the binding in the buffer may have a significant impact and require measurement or prediction of binding properties of a drug.

To obtain the transport flux (J), the accumulated amount was normalized by the surface area (SA) of the cells and the sampling time (t) at each time point:

4 $$\eqalign{ & J\left( {nmol \times c{m^{ - 2}} \times mi{n^{ - 1}}} \right) \cr & = {A_{accumulated}}^{\left( {nmol} \right)}/(SA\left( {c{m^2}} \right) \times t\left( {{\rm{min}}} \right)) \cr} $$

The mean flux was based on time periods of stable flux, i.e., 40–120 min.

The apparent permeability (P) of the drug transport across BECs is estimated by normalizing the flux by the concentration gradient (ΔC).

5 $$\:P\:(cm/min)=\raisebox{1ex}{$J\:\left(nmol\times\:{cm}^{-2}\times\:{min}^{-1}\right)$}\!\left/\:\!\raisebox{-1ex}{$\varDelta\:C\:(nmol/{cm}^{3})$}\right.$$

Where ΔC is the mean measured donor concentration, as the sink condition was assumed. The sink condition was validated by comparing the amount of drug in the donor before and after the transport experiment, with a maximum loss of 20% considered acceptable to ensure no significant change in the concentration gradient.

To assess the intracellular accumulation in the cells of the BEC system, the partition coefficient (K_p,u,cell_) was calculated using the total concentration of the cells (C_tot, cell_) and the concentration of the buffer in the donor compartment. The drug was assumed to be fully unbound in the buffer (C_u, buffer_):

6 $$\:{K}_{p,u,cell}=\raisebox{1ex}{${C}_{tot,cell}$}\!\left/\:\!\raisebox{-1ex}{${C}_{u,buffer}$}\right.$$

To assess the extent of drug transport across the BECs, the ER was estimated from the P of the transport from B-to-A (P_B◊A_) to that in the A-to-B (P_A◊B_) direction.

7 $$\:ER=\raisebox{1ex}{${P}_{B\to\:A}(cm/min)$}\!\left/\:\!\raisebox{-1ex}{${P}_{A\to\:B}(cm/min)$}\right.$$

To be able to compare the extent of drug transport across the BBB in vitro with that obtained in vivo, the unbound partition coefficients, K_p,uu,brain_, were used. In vitro, K_p,uu,brain_ is estimated by inversion of the ER:

8 $$In\>vitro\>{K_{p,uu,brain}} \approx {\raise0.7ex\hbox{$1$} \!\mathord{\left/{\vphantom {1 {ER}}}\right.\kern-\nulldelimiterspace}\!\lower0.7ex\hbox{${ER}$}}$$

Values of in vitro K_p,uu,brain_ above unity indicate predominant active uptake.

#### Culturing immortalized brain endothelial cells

The immortalized human cerebral microvascular endothelial cell line hCMEC/D3 was cultured in EBM^®^-2 medium supplemented with 5% FBS, 1% pen/strep, 1 ng/mL bFGF, 1.4 µM hydrocortisone, 1% chemically defined lipid concentrate, 10 mM HEPES, and 5 µg/mL ascorbic acid. For uptake experiments, 6.7 × 10^4^ cells/well were seeded onto 24-well plates (1.9 cm^2^) pre-coated with collagen type I and used for experiments at approximately 100% confluency (approx. four days later). The cells were used for experiments between passages 14–24.

#### Oxycodone uptake into immortalized brain endothelial cells

Oxycodone uptake into hCMEC/D3 was evaluated at different concentrations and incubation times in transport buffer (10% HBSS in Milli-Q water supplemented with 10 mM HEPES, 0.375% sodium bicarbonate and 0.05% bovine serum albumin (BSA, pH 7.4). Two types of uptake experiments were conducted, (1) time-dependent oxycodone uptake at 200 nM and incubation times of 2, 5, 10, 30, or 60 min, and (2) concentration-dependent oxycodone uptake at concentrations of 5, 50, 200, 1000, or 5000 nM with a fixed incubation time of 5 min. For the concentration-dependent uptake studies, five concentration levels around 200 nM were chosen, and an incubation time of 5 min was selected as the oxycodone uptake rate was linear at this time point. In addition, to explore the impact of oxycodone concentration on the extent of BBB transport in vivo, a rat microdialysis pilot study was performed (Supplementary Materials).

Before the oxycodone uptake experiments, hCMEC/D3 cells were washed using transport buffer. The oxycodone experiment was initiated by incubating the cells with oxycodone at the intended concentration and for the intended incubation time at 37 °C and 90 rpm (orbital shaking). After the incubation, the transport buffer was aspirated and cells were washed with an ice-cold washing buffer (10% HBSS in Milli-Q water supplemented with 0.375% sodium bicarbonate) to halt uptake processes and remove excessive drug. Cells were lysed by incubation in 200 µL lysis buffer for 10 min at room temperature. All samples were stored at -80 °C pending bioanalysis.

Using hCMEC/D3 cells, the rate of drug transport into the cells, i.e., flux, was determined by normalizing the amount of drug in the cells to the surface area and the incubation time. To obtain the drug amount in the cell, the measured concentration was multiplied by an estimated cell volume. To assess the cell volume, the surface area of the well was multiplied by the estimated height of the cell. A microscopically estimated height of 5 μm for hCMEC/D3 was employed to assess the average cell volume. This estimate is higher than the range of values reported for primary rBECs. Hence, the estimated drug amount could be underestimated.

To assess saturable uptake of oxycodone into hCMEC/D3, the concentration-dependency of flux was evaluated by fitting the data to the Michaelis-Menten model:

9 $$\:J=\frac{{J}_{max}\times\left[S\right]}{{K}_{m}+\left[S\right]}$$

Where J is the flux (nmol×cm^− 2^×min^− 1^), J_max_ is maximum flux, i.e., the flux extrapolated to very high substrate concentrations [S] (µM), and K_m_ (µM) is the Michaelis-Menten constant that is the substrate concentration required for the achievement of half-maximum flux.

To assess the intracellular accumulation in hCMEC/D3 cells, the partition coefficient (K_p,u,cell_) was calculated as described above (Eq. 6) using the total concentration of the cells (C_tot, cell_) and the concentration of the virtually protein-free buffer where the drug was assumed to be fully unbound (C_u, buffer_).

#### Equilibrium dialysis to evaluate the extent of oxycodone binding in hCMEC/D3 cells

Equilibrium dialysis was used to measure the unbound fraction of oxycodone in hCMEC/D3 lysate (f_u, cell_) following the procedures previously described [47, 48], with modifications adapted to cell lysates [49]. To obtain blank hCMEC/D3 lysate, hCMEC/D3 was seeded onto 24-well plates as described above. Four days later, the cells were washed using washing buffer and immediately lysed in 200 µL lysis buffer as described above (cells in 0.1% Triton X100 in Milli-Q water, 0.95:200 µL v: v).

Cell lysate spiked with oxycodone with final concentrations of 2000 nM was dialyzed against an equal volume of PBS in a Teflon 96-well plate (Model HTD96b, HTDialysis, Gales Ferry, CT, USA) separated with a regenerated cellulose membrane (molecular weight cutoff 12–14 kDa). Samples were incubated for 6 h at 37 °C at 200 rpm in an incubator with orbital shaking (MaxQ4450 Thermo Fisher Scientific, NinoLab, Sweden).

At the end of the experiment, buffer and tissue samples were collected and matrix-matched for bioanalysis using LC-MS/MS. The matrix matching included PBS sample dilution 1:1 (v: v) with blank hCMEC/D3 lysate, and vice versa, i.e., lysate sample dilution 1:1 in blank PBS. Drug thermostability in the tissues was also evaluated in each experiment. All samples, following dialysis, were stored at -20 °C pending bioanalysis. The fraction of unbound drug (f_u_) in the hCMEC/D3 cells was calculated as follows:

10 $$\:{f}_{u}=\frac{\raisebox{1ex}{$1$}\!\left/\:\!\raisebox{-1ex}{$D$}\right.}{\left(\left(\raisebox{1ex}{$1$}\!\left/\:\!\raisebox{-1ex}{${f}_{u,D}$}\right.\right)-1\right)+\raisebox{1ex}{$1$}\!\left/\:\!\raisebox{-1ex}{$D$}\right.}$$

where D is the dilution factor, which was 211.5 for hCMEC/D3 lysate, based on the estimated cell volume and the lysis buffer volume. f_u, D_ is the fraction of unbound drug in the hCMEC/D3 lysate, calculated as follows:

11 $$\:{f}_{u,\:D}=\raisebox{1ex}{${C}_{u,buffer}$}\!\left/\:\!\raisebox{-1ex}{${C}_{tissue}$}\right.$$

Where C_u, buffer_ is the drug concentration in the PBS compartment and C_tissue_ is the drug concentration in the hCMEC/D3 lysate compartment. In all equilibrium dialysis experiments, samples were collected both before and after the procedure from the spiked tissues. These samples were analyzed to determine relative recovery and thermostability, and the results consistently fell within the acceptable range of 80–120%.

#### Oxycodone uptake into immortalized brain endothelial cells in the presence of interleukin-6

The oxycodone uptake into hCMEC/D3 was performed in the presence of IL-6 to evaluate the cytokine’s impact on oxycodone uptake. The hCMEC/D3 cells were pre-incubated with IL-6 at concentrations of 1, 10, and 100 ng/mL, for 60 min prior to the uptake study. The time and concentrations were selected based on the protocol published by de Vries et al., who observed a significant decrease in rBEC TEER already within 60 min [50]. Thereafter, hCMEC/D3 was incubated with 200 nM oxycodone for 5 min as described above.

Cell viability was assessed for all conditions included in the IL-6 experiments, using (i) dead cell staining by Erythrosine B, (ii) the XTT Cell Proliferation Kit II (sodium 3´-[1- (phenylaminocarbonyl)- 3,4- tetrazolium]-bis (4-methoxy6-nitro) benzene sulfonic acid hydrate (XTT) based colorimetric assay, Roche, Sigma-Aldrich, Saint Louis, USA), (iii) the LDH Cytotoxicity Detection Kit (lactate dehydrogenase (LDH) colorimetric assay, Roche, Sigma-Aldrich, Saint Louis, USA).

To visualize the cell viability, dead cells were stained using Erythrosine B [51]. hCMEC/D3 cells were seeded on a separate 96-well plate (Corning^®^) and treated as in the uptake experiment. Thereafter, the cells were incubated with 100 µL of 0.1% Erythrosine B staining solution in PBS for 2 min. The dye was then aspirated, and cells were washed with transport buffer until clear. Stained cells were visualized in the presence of 100 µL blank transport buffer and imaged using a Leica Microsystem DMi8 automated (Leica, Wetzlar, Germany). Controls included untreated cells (live cell control), and cells incubated with 0.01% Triton X-100 in Milli-Q water (dead cell control).

For the XTT assay, hCMEC/D3 cells were seeded on a separate 96-well plate (Corning^®^) and treated as in the uptake experiment. To terminate the uptake experiment, cells were washed using washing buffer. XTT labeling reagent and an electron coupling reagent were thawed and mixed according to the manufacturer’s instructions. A final concentration of 0.3 mg/mL XTT labeling mixture in washing buffer was added to each well and incubated at 37 °C for 2 h. The absorbance of the formazan product was measured at 466 nm with a reference wavelength of 650 nm using a Spectrostar Nano plate reader (BMG Labtech, Ortenberg, Germany). Controls included blank transport buffer without cells (background), untreated cells (live cell control), and cells incubated in Milli-Q water (dead cell control).

For the LDH assay, transport buffer from oxycodone and IL-6-treated and control hCMEC/D3 cells was collected at the time of the uptake experiment and stored at -80 °C until analysis. The LDH assay was performed onto an optically clear flat-bottom microplate (Corning^®^ 96-well plate) by incubating 100 µL of cell-free transport buffer from the experiments (10% HBSS in Milli-Q water supplemented with 10 mM HEPES, 0.375% sodium bicarbonate and 0.05% BSA, pH 7.4) with 100 µL of the reaction mixture for 15 min at 37 °C, according to the manufacturer’s instructions. As the LDH kit solutions are light sensitive, the experiment was performed protected from light. Absorbance was measured at 492 nm with a reference wavelength of 690 nm using a Tecan Spark^®^ Multimode Microplate Reader (Tecan Group Ltd., Männedorf, Switzerland). Controls included blank transport buffer without cells (background control), untreated cells (live cell control), and cells incubated with 2% Triton X-100 (dead cell control).

To obtain the relative viability of the cells in each well, the experimental (Exp) value obtained by the XTT or LDH assays was adjusted with values from the respective dead and live cell controls:

12 $$\eqalign{ Viability\>\left( \% \right) = & (Exp\>value - Dead\>cell\>control) \cr & /(Live\>cell\>control - Dead\>cell\>control) \cr} $$

### Brain parenchymal cell uptake and binding

The cellular uptake of antiporter substrates was compared between brain endothelial and parenchymal cells. Uptake into brain parenchymal cells and binding were evaluated using the rat brain slice method and equilibrium dialysis.

#### Animals

Sprague-Dawley rats of 250–300 g (Taconic, Lille Skensved, Denmark), were housed in groups under controlled conditions of 20–22 °C and 45–65% humidity. The housing environment followed a 12-hour light/dark cycle, and the rats were provided with free access to food and water. The rats were allowed a seven-day acclimatization period to adjust to the new environment before the commencement of the experiments. To obtain the brains required for the performance of equilibrium dialysis and brain slice assay, the rats were anaesthetized using inhalation of 5% isoflurane, decapitated, and the brains were isolated and stored at -20 °C pending equilibrium dialysis. For brain slice assay, the brains were quickly transferred onto a pre-oxygenated blank artificial extracellular fluid (aECF, pH 7.6 at room temperature) on ice and used for an experiment on the same day.

#### Equilibrium dialysis to evaluate the extent of oxycodone binding in rat brain

Equilibrium dialysis was performed to measure the unbound fraction of oxycodone in the rat brain (f_u, brain_) as described for hCMEC/D3 lysate above. For the experiments, brain tissue homogenate 1:9 (w: v) in phosphate-buffered saline (PBS, pH 7.4) was prepared at final oxycodone concentrations of 1000 nM. Samples were collected and handled as described above. The dilution factor of brain tissue, used in Eq. 10, was 10.

#### Brain slice assay for assessment of oxycodone brain parenchymal cell uptake

Oxycodone intra-brain distribution, including brain tissue binding and intracellular uptake, was evaluated using the brain slice assay by measuring the unbound volume of distribution in the brain, V_u, brain_ (mL/g brain), based on established protocols [24, 25]. Briefly, six 300 μm brain coronal slices from the rostral striatum area (*N* = 3 rats) were prepared using a Leica VT1200 microtome slicer (Leica Microsystems AB, Sweden). These slices were incubated in an Ø80-mm flat-bottomed glass beaker containing pre-oxygenated aECF with a final drug concentration of 100 nM. To evaluate the impact of oxycodone concentration on intra-brain distribution, brain slices were incubated at concentrations of 500, 1000, and 5000 nM. The beaker was covered with a custom lid fitted with Teflon fluorinated ethylene-propylene film (Teflon FEP film 50 Å, 12.7 μm thickness) to allow air exchange while preventing buffer evaporation. Incubation was carried out at 37 °C for 5 h at 45 rpm in an orbital shaker (MaxQ4450) with a constant oxygen flow of 75–80 mL per minute. The pH of the aECF was measured both at the beginning and after incubation. Post-incubation, each brain slice was dried on filter paper, weighed, and homogenized with aECF in a 1:9 ratio (w: v). Buffer was sampled in duplicates directly from the glass beaker and mixed with an equivalent volume of blank brain homogenate (1:4, w: v with aECF) to match the matrix for bioanalysis. The thermostability of the compounds at 37 °C was assessed in parallel aECF solutions without brain slices. All samples were stored at -20 °C pending UPLC-MS/MS analysis.

To assess the extent of intracellular accumulation in brain parenchymal cells, the total brain slice-to-unbound buffer concentration ratio, i.e., V_u, brain_ (mL×g brain^− 1^) was estimated from data obtained using the brain slice method. V_u, brain_ was calculated as the ratio of the drug amount in the brain slice (A_brain_, nmol×g brain^− 1^=1000 nM) to the terminal concentration in the aECF (C_u, buffer_, nmol×mL^− 1^=1000 nM), which assumes that equilibrium was achieved within the incubation time and that the aECF and in the ISF of the brain slices are equivalent at equilibrium:

13 $$\:{V}_{u,brain}=\frac{{A}_{brain}-{V}_{i}\times\:{C}_{u,buffer}}{{C}_{u,buffer}\times\:\left(1-{V}_{i}\right)}$$

Where V_i_ is the volume of the remaining aECF layer around the brain slices, which was estimated to 0.133 mL×g brain^− 1^ using ^14^C-sucrose [52]. The brain tissue density was assumed to be 1 g/mL.

### Assessment of the extent of cellular barrier transport

An overview of plasma and brain compartments and methods to study the extent of drug membrane transport is presented in Fig. 1. To assess the extent of transport across the brain endothelial (hCMEC/D3) and parenchymal cell barriers (rat brain slice), K_p,uu,cell_ was obtained from endothelial K_p,u,cell_ and parenchymal V_u, brain_ by adjusting for the drug binding to hCMEC/D3 and brain tissue, respectively, as described for parenchymal cells previously [23]:

14 $$\:{K}_{p,uu,cell}={K}_{p,u,cell}\times{f}_{u,cell}$$

and

15 $$\:{K}_{p,uu,cell}={V}_{u,brain}\times{f}_{u,brain}$$

The standard deviation (SD) for K_p,uu,cell_ was calculated according to the law of propagation of error, as it was derived from two parameters with uncertainties [53]. The propagation of uncertainty of the product (f), i.e., K_p,uu,cell_, was estimated according to the following equation, where σ_A_ and σ_B_ are the SD of K_p,u,cell_ or V_u, brain_ and f_u, cell_ or f_u, brain_, respectively.

16 $$\:{\sigma\:}_{f}\approx\:\:\left|f\right|\times\:\sqrt{{\left(\frac{{\sigma\:}_{A}}{A}\right)}^{2}+{\left(\frac{{\sigma\:}_{B}}{B}\right)}^{2}+2\frac{{\sigma\:}_{AB}}{AB}}$$

The covariance σ_AB_ was calculated with the correlation (r) as σ_AB_ = rσ_A_σ_B_. The innate correlation between variables were assumed to be *r* = – 0.5.

### Bioanalysis

#### Bioanalysis of oxycodone by UPLC-MSMS

Quantitative analysis of oxycodone in samples, matrix-matched standards, quality control (QC) samples and the respective blanks, across various biological matrices including cell media, cell lysate, and brain tissue homogenate, was conducted using ultraperformance liquid chromatography-tandem mass spectrometry (UPLC-MS/MS). Samples containing oxycodone and oxycodone-D6 (used as the oxycodone internal standard, IS) were prepared and quantified using a previously described method [12].

Calibration curves were constructed in transport buffer or differentiation media for samples collected from hCMEC/D3 uptake experiments, hCMEC/D3 equilibrium dialysis, and primary BEC transport experiments, at concentrations of 1-5000 nM, and in brain tissue homogenate for samples collected from equilibrium dialysis (1:19, w: v, in PBS) and brain slice assay (1:9, w: v, in PBS) at concentrations of 1-3200 nM. QC samples were prepared at four different concentration levels evenly spread out across the calibration curve ranges for all matrices. Calibration curves were generated using linear regression with a 1/X² weighing function, achieving an R^2^ ≥ 0.99. The lower limit of quantification (LLOQ) was set to the lowest standard (1 nM). Blank matrix samples with and without IS were included in each run.

##### Sample preparation

Cell lysate and cell media samples were thawed, gently vortexed, and precipitated 1:3 in acetonitrile spiked with IS (8 ng/mL) on 96-well plates. Samples were then vortexed and centrifuged at 10,000 rpm for 5 min. After centrifugation, the supernatant was transferred to new 96-well plates, diluted 1:2 in Milli-Q water, and vortexed. Brain homogenate samples were precipitated 1:2 in acetonitrile spiked with IS, vortexed, centrifuged at 10,000 rpm for 5 min, and the supernatants were transferred to microvials placed on a 96-well plate, diluted 1:2 in Milli-Q water and vortexed. All prepared samples were placed on a 96-well plate before the 5 µL injection onto the UPLC column.

##### Quantification

The quantification was conducted using an Acquity Ultra-Performance Liquid Chromatography instrument coupled to a Xevo TQ-S Micro mass spectrometer (Waters Corporation, Milford, Massachusetts, USA). The separation was achieved on an AQUITY UPLC BEH C18-column (1.7 μm, 2.1 × 50 mm) coupled with a VanGuard Pre-Column made of the same material (Waters Corporation, Milford, Massachusetts, USA). The compounds of interest were separated using an elution gradient of the mobile phases of 0.1% formic acid in Milli-Q water and in acetonitrile, respectively. The flow rate was 0.3 mL/min. Ionization was performed using an Electro Spray Ionization probe in positive mode. Multiple reaction monitoring (MRM) mode was used with the following transitions monitored: oxycodone (316.11 → 298.1 m/z) and oxycodone-D6 (322.18 → 304.1 m/z). The software used for quantification of analytes was MassLynx version 4.2 and TargetLynx (Waters Corporation, Milford, Massachusetts, USA).

#### Bioanalysis of ^3^H-pyrilamine

^3^H-pyrilamine was quantified in cells and cell media samples from the BEC transport studies. Immediately after the experiment, the samples were mixed with 2 mL of Ultima Gold 241 TM in scintillation vials and analyzed using a Tri-Carb 2910 TR Liquid Scintillation Analyzer (Perkin Elmer, MA, USA).

### Exclusion criteria and statistics

Samples below LLOQ or identified as outliers were excluded in calculations and statistical analysis of data. Outliers were identified using the robust regression and outlier removal method in GraphPad.

Statistical analyses were conducted using GraphPad Prism version 9.0.0 for Windows (GraphPad Software, San Diego, California, USA). Two-tailed t-tests were used for comparisons of permeabilities between different conditions, as well as between transport directions in the BEC transport studies. A mixed-effects analysis with the Geisser-Greenhouse correction and Tukey’s multiple comparisons test was performed for comparisons between different oxycodone incubation times and concentrations in hCMEC/D3 uptake experiments. Oxycodone flux data conducted at varying concentrations in hCMEC/D3 were fitted to the Michaelis-Menten model. To compare oxycodone hCMEC/D3 K_p,uu,cell_ across different incubation times and concentrations, oxycodone uptake into hCMEC/D3 under varying IL-6 concentrations, and the concentration-dependency of oxycodone uptake into brain slices, ordinary one-way analysis of variance (ANOVA) tests followed by Tukey’s multiple comparisons tests were used. For comparisons of oxycodone hCMEC/D3 K_p,uu,cell_ between control and IL-6-treated cells, an ordinary one-way ANOVA with Dunnett’s multiple comparisons test was employed. To assess the viability of hCMEC/D3, as determined by XTT and LDH assays, between control and IL-6 pre-treated conditions, repeated measures one-way ANOVA with the Geisser-Greenhouse correction and Dunnett’s multiple comparisons test was utilized. Differences were considered significant at *p* < 0.05. N indicates number of biological replicates, while n indicate number of technical replicates.

## Results

### Active uptake into and across primary brain endothelial cells

**Fig. 2:**
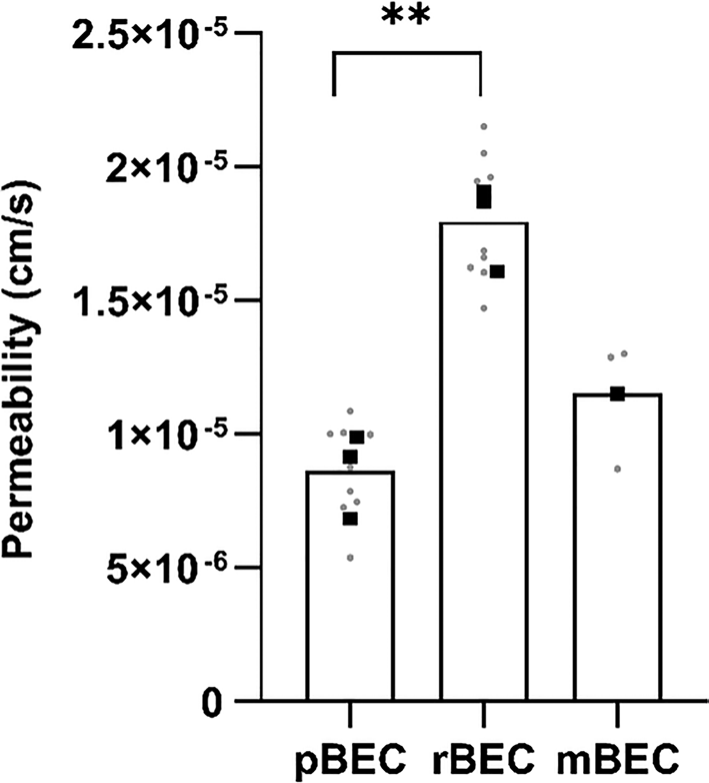
Oxycodone transport rate described by permeability across primary brain endothelial cells (BECs) of different origins. Apparent permeability (cm/s) of oxycodone (200 nM) across primary pig (pBEC, *N* = 3, *n* = 3), rat (rBEC, *N* = 3, *n* = 3), and mouse (mBEC, *N* = 1, *n* = 3) BECs, from apical to basolateral compartments. Batch means are indicated by black squares and technical replicates by gray dots. The statistical comparison between groups was performed using an unpaired two-tailed t-test, **p = 0.0021

**Fig. 3:**
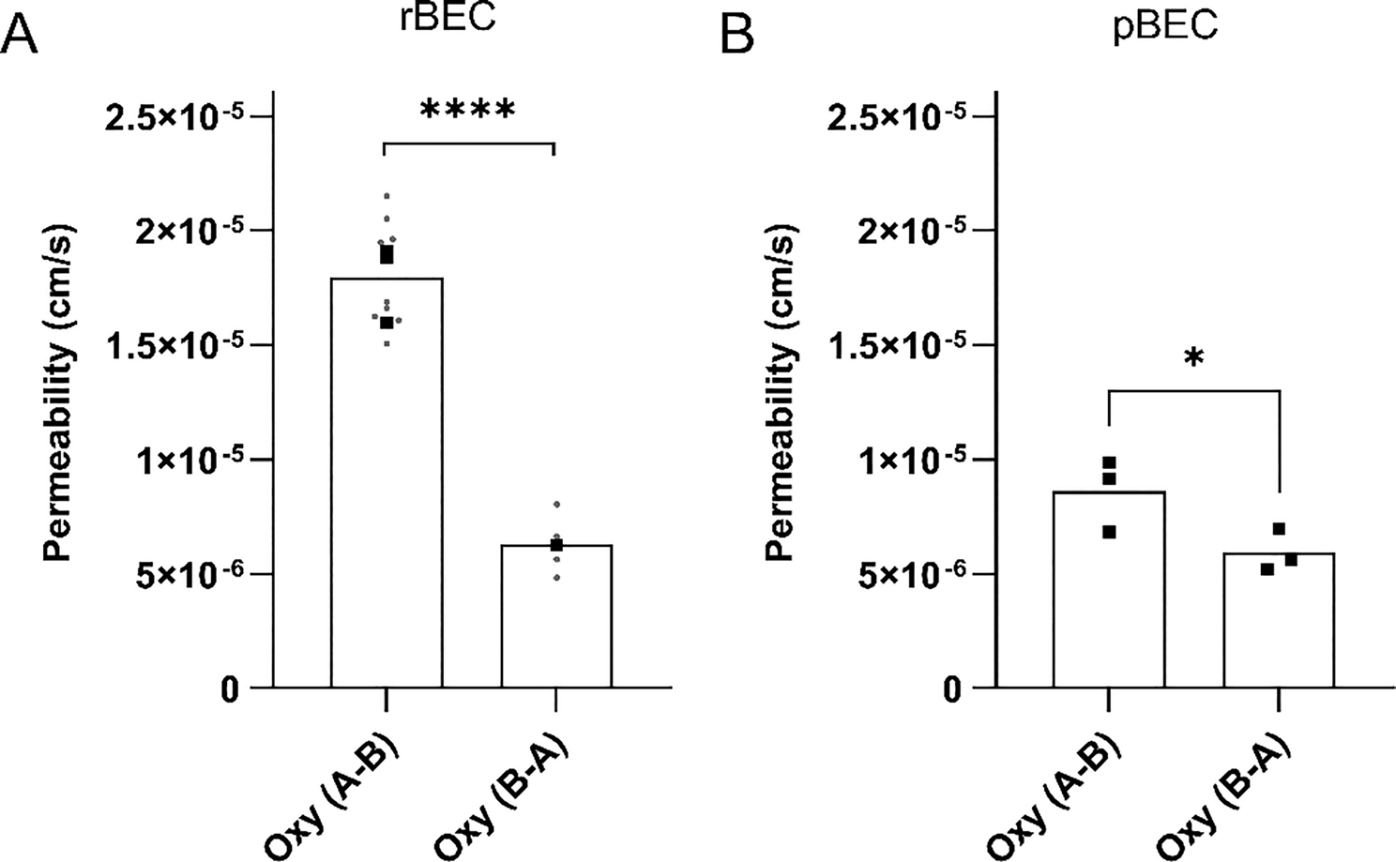
Oxycodone permeability in both transport directions across rat (3**A**) and porcine (3**B**) primary brain endothelial cells (BECs). Oxycodone (Oxy, 200 nM) transport rate described by apparent permeability (cm/s). The statistical comparison between transport directions across primary rat BECs (rBEC), from apical to basolateral (A-to-B) (*N* = 3, *n* = 3) and B-to-A (*N* = 1, *n* = 4) compartments, was performed on technical replicates using an unpaired two-tailed t-test (****, *p* < 0.0001). The statistical comparison between transport directions across primary pig BECs (pBEC), from A-to-B (*N* = 3, *n* = 3) and B-to-A (*N* = 3, *n* = 3) compartments was performed on biological replicates using a paired two-tailed t-test (*p = 0.04). Batch means are indicated by black squares, and technical replicates by gray dots (only in 3 A)

To assess the rate (measured by permeability) and extent (measured by the cell-to-buffer concentration ratio) of oxycodone and ^3^H-pyrilamine transport into and across primary BECs and to compare with in vivo data, transcellular transport studies were performed at clinically relevant drug concentrations. In all experiments, the drug was assumed to be fully unbound in the virtually protein-free buffer. The mean TEER across the cells after correcting for the mean blank filter TEER was 343 ± 174 Ω × cm^2^, 42 ± 24 Ω × cm^2^, and 115 ± 19 Ω × cm^2^, across pBEC, rBEC, and mBEC, respectively (Table S1-S3). Transendothelial oxycodone transport was studied and observed across pBEC, rBEC, and mBEC monolayers, while ^3^H-pyrilamine transport was studied and observed across rBECs. The apparent permeability of the canonical H^+^/OC antiporter substrate ^3^H-pyrilamine across the rBEC in the transcellular set-up from A-to-B, at an incubation concentration of 9 nM, was 1.0 ± 0.1 × 10^− 5^ cm/s (*N* = 3, *n* = 3). The apparent permeability of oxycodone across the pBEC and rBEC in the transcellular set-up from A-to-B, at an incubation concentration of 200 nM, was 0.9 ± 0.2 × 10^− 5^ cm/s (*N* = 3, *n* = 3), and 1.8 ± 0.2 × 10^− 5^ cm/s (*N* = 3, *n* = 3), respectively (Fig. 2). This indicates that the oxycodone permeability is higher across rBECs than across pBECs. In the mBEC pilot study, the apparent oxycodone permeability was 1.2 × 10^− 5^ cm/s (*N* = 1, *n* = 3, A-to-B, at 200 nM concentration, Fig. 2). The oxycodone permeability A-to-B was higher than that from B-to-A across both rBEC and pBEC (Fig. 3A-B), suggesting active transport across the two BBB cell models. The oxycodone ER across rBECs was estimated to 0.4, resulting in an estimated in vitro K_p,uu,brain_ of 2.4, obtained by 1/ER. The oxycodone ER across pBECs was estimated to 0.7 ± 0.1, with a corresponding in vitro K_p,uu,brain_ of 1.5 ± 0.2. The obtained cellular pharmacokinetic parameters further support that oxycodone exhibits an active uptake across both rBECs and pBECs in the transcellular set-up.

The extent of oxycodone uptake into BEC was initially determined by K_p,u,cell_, i.e., total cell-to-unbound media concentration ratio (Eq. 6). The oxycodone K_p,u,cell_ in pBECs was 18.6 ± 11.1 (*N* = 3, *n* = 1–2). Oxycodone K_p,u,cell_ in pBECs was variable with a coefficient of variation (CV) of 59%. Also, oxycodone cell concentrations were below LLOQ for a number of pBEC replicates. Due to the same reason, the extent of oxycodone uptake into rBECs and mBECs could not be assessed. The ^3^H-pyrilamine K_p,u,cell_ measured in the rBEC transcellular set-up was 38.5 ± 19.5 (*N* = 3, *n* = 3), indicating an intracellular accumulation of ^3^H-pyrilamine in the rBECs, also with a large variability (CV of 51%).

### Oxycodone uptake into immortalized brain endothelial cells in normal and interleukin-6 treated conditions

**Fig. 4:**
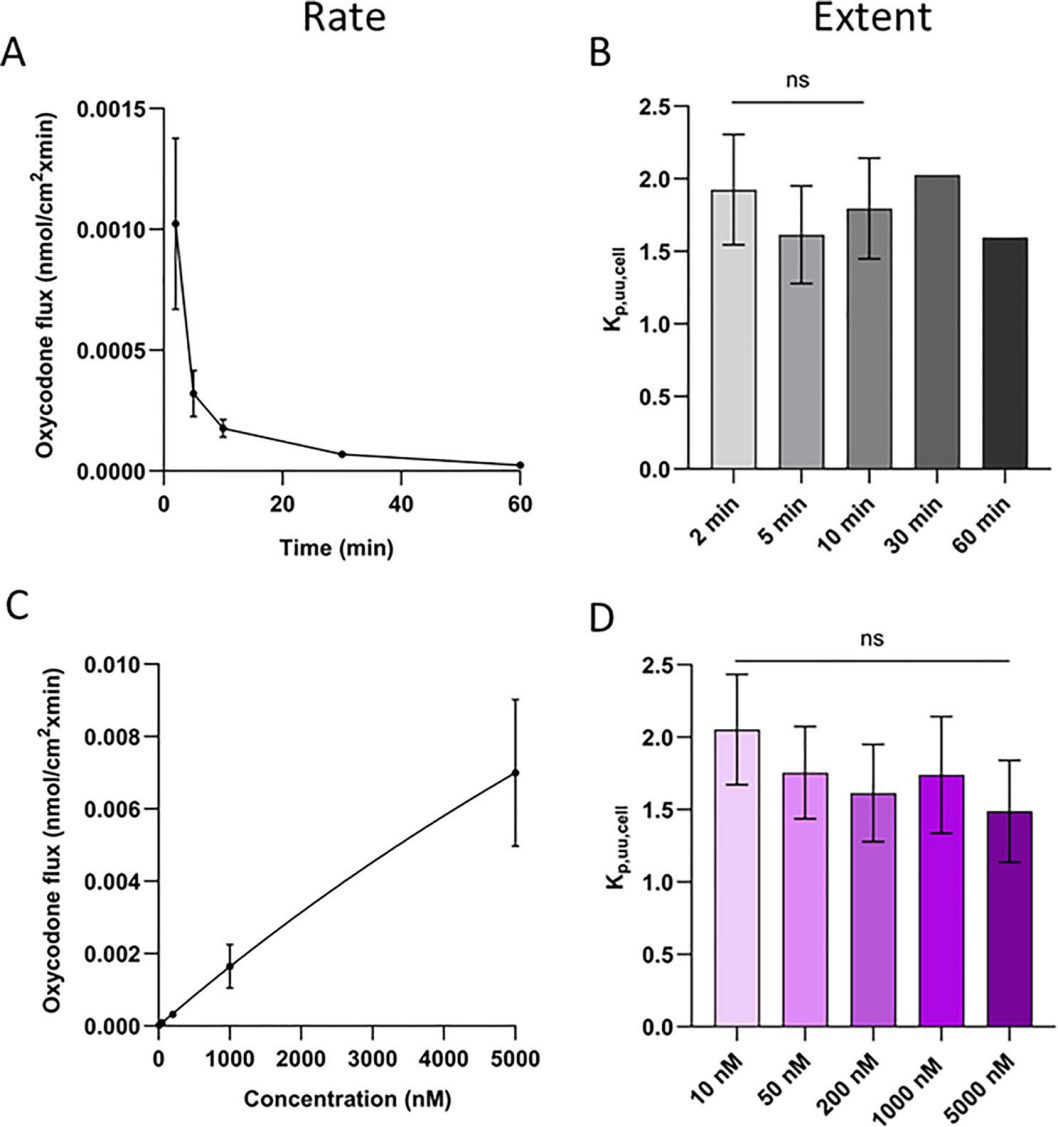
Rate (**A** and **C**) and extent (**B** and **D**) of oxycodone uptake into hCMEC/D3. **A**) Flux at different time points (*N* = 1–5, *n* = 3 per incubation time and passage), **B**) K_p,uu,cell_ at different time points (*N* = 1–5, *n* = 3 per incubation time and passage), **C**) Flux at different concentrations fitted to the Michaelis-Menten model, **D**) K_p,uu,cell_ at different concentrations. The oxycodone uptake over time was performed at 200 nM. The oxycodone uptake across concentrations was performed using a 5-minute incubation time. For comparisons between different oxycodone incubation times and concentrations, a mixed-effects analysis with the Geisser-Greenhouse correction and Tukey’s multiple comparisons test was performed. To compare oxycodone K_p,uu,cell_ across different incubation times and concentrations, an ordinary one-way ANOVA test followed by Tukey’s multiple comparisons test was used. Oxycodone flux data conducted at varying concentrations were fitted to the Michaelis-Menten model. Statistics are shown on the group level

To evaluate the rate (i.e., flux) and extent (i.e., cell-to-buffer concentration ratios K_p,u,cell_ and K_p,uu,cell_) of oxycodone uptake into endothelial cells at a clinically relevant concentration range, uptake studies in hCMEC/D3 were performed at concentrations of 10–5000 nM (Fig. 4). The rate of uptake presented as flux was both time- and concentration-dependent (Fig. 4A and C). J_max_ was estimated to 0.039 nmol/min/cm^2^, and K_m_ to 22.6 µM. On the contrary, the extent of uptake was both time- and concentration-independent (Fig. 4B and D), with a mean oxycodone total cell-to-unbound cell media concentration ratio (K_p,u,cell_) of 16.6 ± 3.2 across all times and concentrations (*N* = 5, *n* = 12–15 per passage). To obtain information about the transport at the cellular barrier, K_p,u,cell_ was corrected for non-specific binding to cellular components using f_u, cell_ obtained by equilibrium dialysis of blank hCMEC/D3 lysate and the propagation of error model (Eqs. 14 and 16), to estimate endothelial K_p,uu,cell_. The oxycodone f_u, cell_ was 10.5 ± 2.2% (*N* = 1, *n* = 6). Consequently, the oxycodone unbound cell-to-cell media concentration ratio K_p,uu,cell_ in hCMEC/D3 was 1.7 ± 0.3.

**Fig. 5:**
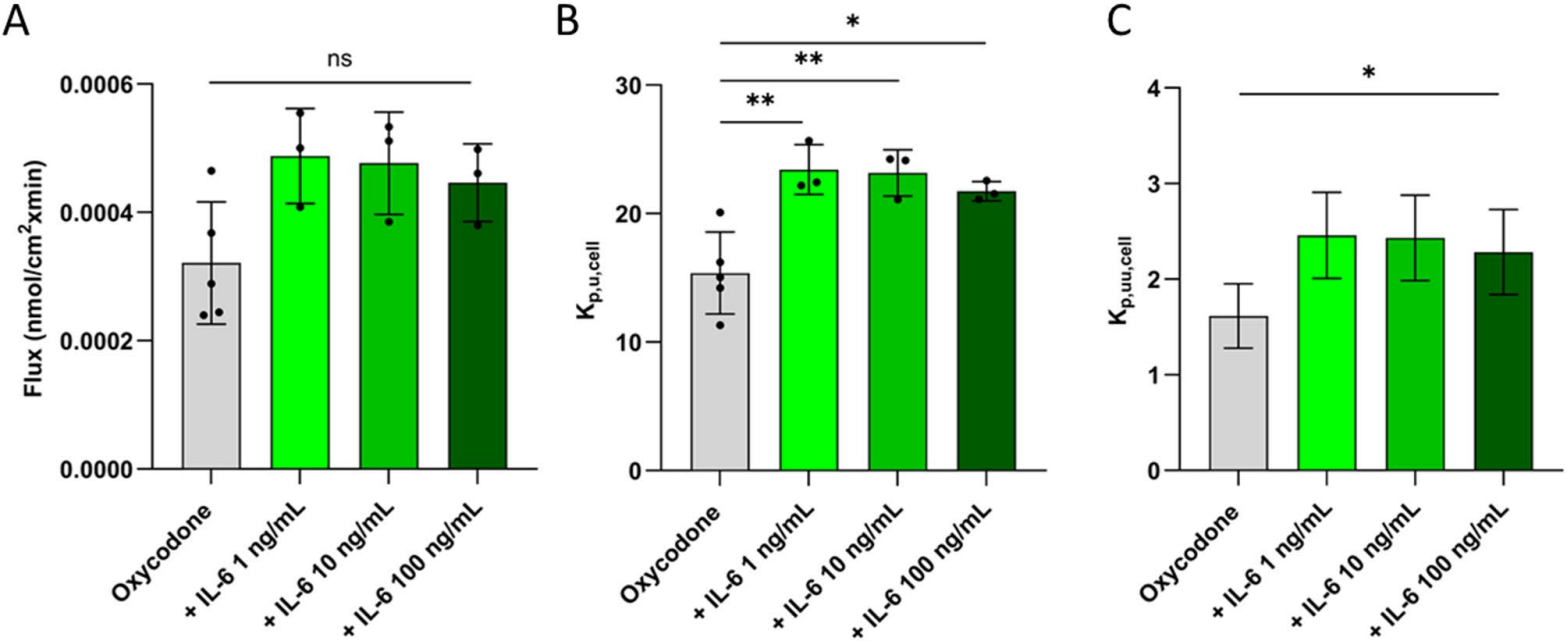
Rate and extent of oxycodone uptake into hCMEC/D3 in the absence and presence of interleukin-6 (IL-6). Oxycodone (200 nM) uptake into hCMEC/D3 after 5 min incubation in the absence and presence of IL-6, 60-minute pre-treatment at different concentrations (1, 10, 100 ng/mL). **A**) Rate of uptake presented as flux, **B**) Extent of uptake presented as total cell-to-unbound media concentration ratio (K_p,u,cell_), and **C**) Extent of uptake presented as unbound cell-to-media concentration ratio (K_p,uu,cell_) obtained by K_p,u,cell_ and f_u, cell_ using the propagation of error calculation, assuming that the IL-6 treatment did not alter f_u, cell_. To compare oxycodone uptake into hCMEC/D3 under varying IL-6 concentrations, an ordinary one-way ANOVA test followed by Tukey’s multiple comparisons test or Dunnett’s multiple comparisons test was used. Further statistical test details are presented in Table S4

To evaluate the impact of IL-6 on oxycodone uptake into endothelial cells, hCMEC/D3 cells were used in the presence of IL-6 at concentrations of 1, 10, and 100 ng/mL. The XTT and LDH assays showed that the 60-minute IL-6 treatment did not affect the viability of the hCMEC/D3 at investigated concentrations, as IL-6 treated cells exhibited similar viability as the live cell controls (Fig. S1). In the XTT assay, the mean viability was around 100% for all oxycodone and/or IL-6-treated cells, without any differences compared to live cell control. The LDH assay showed mean viability in each group above 75% compared to the control, yet with higher intra-group variability. Erythrosine B staining of hCMEC/D3 further supported the lack of cytotoxicity in the oxycodone and/or IL-6-treated conditions, as treated cells exhibited similar morphology to live cells (Fig. S1). Differences in the rate of oxycodone uptake (flux) did not reach significance between control and IL-6 treated cells (Fig. 5A, *p* > 0.05). The extent of uptake was higher in IL-6 treated cells compared to control, but similar between the different IL-6 concentrations (Fig. 5B-C, Table S4), with a mean oxycodone K_p,u,cell_ of 22.8 ± 1.6 (*N* = 3, *n* = 9 per passage). This was on average 1.5-fold higher than the K_p,u,cell_ in control cells. Assuming that IL-6 is not altering non-specific binding to cellular components (f_u, cell_), the mean oxycodone K_p,uu,cell_ in IL-6 treated hCMEC/D3 was 2.4 ± 0.4 (Fig. 5C). The accumulated amount of oxycodone in hCMEC/D3 treated with 200 nM oxycodone and IL-6 ranged from 1.9 to 2.8 pmol/cm^2^, which was slightly higher but without reaching significance compared to 1.6 ± 0.5 pmol/cm^2^ in controls (*p* > 0.05).

### Oxycodone concentration-independent uptake into brain parenchymal cells

To evaluate the oxycodone uptake in brain parenchymal cells, brain slice and equilibrium dialysis assays were performed. Intracellular accumulation of oxycodone was observed with a V_u, brain_ of 3.6 ± 0.1 mL/g brain at 100 nM (*n* = 9). The V_u, brain_ estimation from brain slice studies, corresponds to the K_p,u,cell_ estimation from the cell culture studies, as they are both total tissue-to-unbound buffer concentration ratios at equilibrium. To assess the extent of drug uptake at the brain parenchymal cell barrier, V_u, brain_ was combined with brain tissue binding data using the propagation of error model to assess parenchymal K_p,uu,cell_ (Eqs. 15–16). The f_u, brain_ of oxycodone was 43.0 ± 7.9% (*n* = 6).

**Fig. 6:**
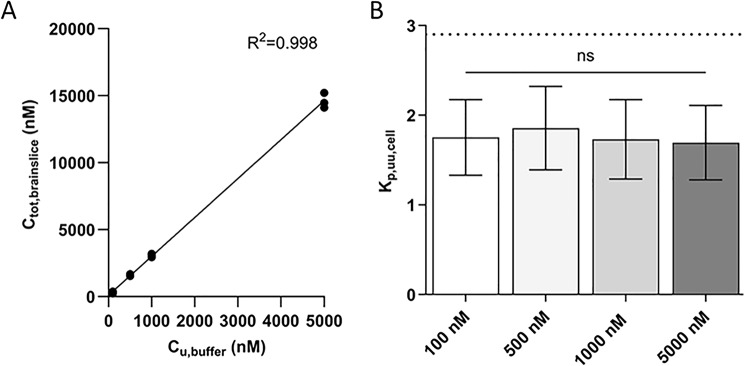
Oxycodone brain parenchymal cell uptake obtained by the brain slice assay. **A**) Correlation between total oxycodone concentration in the brain slice (C_tot, brainslice_, pmol/g brain = nM) and the corresponding initial buffer concentration (nM). Data points represent mean brain slice concentration values from each biological replicate, and the line represents the best-fit linear regression. **B**) K_p,uu,cell_ at 100, 500, 1000 and 5000 nM oxycodone obtained by V_u, brain_ (brain slice, *N* = 3–6 per concentration) and f_u, brain_ (equilibrium dialysis, *N* = 3, *n* = 1–2) values using the propagation of error model for estimation of uncertainty around the mean K_p,uu,cell_ (Eqs. 15–16). The dotted line represents the K_p,uu,cell_ of 2.9 previously estimated by the mathematical pH partition model accounting for pKa value of oxycodone and the volumes of compartments having respective pH values [74]. Comparisons were performed using an ordinary one-way ANOVA followed by Tukey’s multiple comparisons test

To investigate the concentration-dependency of parenchymal cell uptake, the brain slice assay was performed at oxycodone concentrations of 100–5000 nM. The oxycodone unbound buffer concentration and the total concentration in brain slices showed a strong positive linear relationship, suggesting that the uptake of oxycodone by brain slices increases proportionally with buffer concentration (R^2^ > 0.998, Fig. 6A). The intra-brain distribution of oxycodone was thus similar within the concentration range studied, with the mean V_u, brain_ values around 4 mL/g brain across all investigated concentrations (Table S5). Corrected for non-specific binding, the parenchymal K_p,uu,cell_ was 1.7–1.9 across all concentrations (Fig. 6B).

### Comparisons between oxycodone uptake at brain endothelial versus brain parenchymal cells

As described above, a concentration-independent extent of uptake of oxycodone at the brain endothelial cell membrane was observed in hCMEC/D3 (Fig. 4D). Similarly, the extent of uptake at the parenchymal cell membrane was concentration-independent (Fig. 6B), with values between 1.7 and 1.8, which are very similar to that of 1.7 observed in endothelial cells (hCMEC/D3).

To explore if the concentration-independent extent of uptake was also observed across the BBB in vivo, a microdialysis pilot study was performed in a rat (Supplementary Material). The extent of oxycodone transport remained steady across unbound plasma concentrations of 95–8022 nM (i.e., 30–2530 ng/mL, Fig. S2). The unbound concentrations in blood and brain at the three different oxycodone infusion rates resulted in a K_p,uu,brain_ of 5.9 ± 0.2 (CV of 3.5%).

## Discussion

Our study provides significant insights into the in vitro brain disposition of antiporter substrate oxycodone and its relevance to in vivo brain drug delivery. The contribution of the present work is the comparative and quantitative assessment of oxycodone uptake across several species-derived primary BEC models, the immortalized hCMEC/D3 line, and parenchymal brain slices, all studied at clinically relevant concentrations and linked by the same cellular pharmacokinetic metrics (K_p,uu,cell_ and ER). No prior study has systematically connected in vitro and in vivo oxycodone data across species using these translational readouts. The impact of IL-6 treatment on the uptake into hCMEC/D3 cells was also examined. To our knowledge, this is one of the few transport studies evaluating antiporter substrate transport across primary BECs in relation to species-matched in vivo data.

The findings suggest an active net uptake into endothelial cells and to the brain ISF mimicking compartment in BBB cell models of human (hCMEC/D3), pig (pBEC), and rat (rBEC) origin, indicating functional similarities in the H^+^/OC antiporter system across the models and cell origin. In particular, cellular pharmacokinetics data demonstrate active transport of oxycodone across pBECs and rBECs, shown by ER values of 0.7 and 0.4, respectively. This corresponds to in vitro K_p,uu,brain_ of 1.5 and 2.4, respectively, which are consistent with in vivo K_p,uu,brain_ of 2.5 in pigs [13], and 3–4.4 in rats [10, 12], being all above unity. The evidence of a net uptake of oxycodone across cell monolayers appears to be somewhat unique in BECs, as for example, in comparison with a Caco-2 transport study reflecting intestinal drug absorption, the oxycodone ER was estimated to 2 [54], suggesting net efflux. This study by Hassan et al., along with others, suggests that oxycodone may interact with the efflux transporter P-glycoprotein (P-gp) [54–56], although in our primary BECs active influx appeared to dominate. Energy-depletion validation, as well as knock-out of suggested proteins involved in the antiporter system (TM7SF3 and LHFPL6 [2]), would have strengthened mechanistic certainty, however, the convergence of rate- and extent-based metrics supports the notion of active transport.

In this study, the ER is based on permeabilities specific for this system (A-to-B, B-to-A), which include permeabilities across both the BEC layer and the coated cell culture inserts, which were not corrected for paracellular routes. This choice was related to the fact that the focus was on the comparative extent of vectorial transport (ER < 1) rather than absolute intrinsic permeability. It is, therefore, important to note that the permeabilities do not reflect that across the BEC layer itself. To isolate the BEC layer permeability, it is necessary to correct the measured permeability values for permeabilities across both blank filters and the unstirred water layer [57–59]. Using a blank filter for permeability measurements as a baseline control has its limitations, and might be misleading. Transport across permeable supports with cells will be transcellular and the cell layer can limit vectorial water flow due to gravity from the upper to lower chamber (i.e., A-to-B). Transport across cell-free permeable supports can be influenced by vectorial water flow. Accordingly, we observed that the blank filter permeability in the A-to-B direction differs from that in the B-to-A direction, with a notably faster A-to-B transport rate (unpublished observations). However, this directional difference in filter contribution may not persist when cells are present. Consequently, correcting for direction-specific blank filter permeability compared with applying the A-to-B blank filter correction to both directions, could lead to different interpretations of the data. This raises an important question: is it necessary to isolate the intrinsic permeability of the BEC layer, or is the apparent permeability, reflecting the overall system, sufficient for drawing meaningful conclusions? Here, we demonstrate that monoculture (rBEC, pBEC) transcellular transport systems effectively captured the active brain uptake of oxycodone observed in vivo for the respective species, albeit to a slightly lower extent. Importantly, the lower extent of oxycodone brain uptake observed in pigs compared to rats was mirrored in pBECs versus rBECs, with the in vitro K_p,uu,brain_ being approximately 60% of that observed in vivo, for both species. Though the primary BBB cell models have many in vivo-like characteristics, and is highly useful for quantitative and mechanistic studies, it still lacks the interplay with other cells in the neurovascular unit as well as the influence of shear stress and its mechanobiology derivative effects. Differences between our in vivo and in vitro data is therefore expected. Despite this, the species-specific properties of the cells in regard to active uptake are retained in vitro, highlighting the translational potential of these models in reflecting interspecies differences in brain drug transport. Functional species differences of primary BECs represent an important subject for future research, both for studying the inter-species translation of antiporter-mediated transport in vitro, and for advancing the development of brain-targeting drugs. The grounding work in this matter has already been presented by Kurosawa et al. [2], proposing TM7SF3 and LHFPL6 as being involved in antiporter-mediated transport in hCMEC/D3 cells.

TEER was monitored before and after each experiment and confirmed that barrier integrity was maintained. The measured mean TEER value of 42 Ω × cm^2^ in the rat BECs was variable (CV >50%), and lower than previously reported values for primary rat BECs, ranging between 100 and 300 Ω × cm^2^ [46]. In contrast, the mean TEER values of 115 Ω × cm^2^ (mBEC) and 343 Ω × cm^2^ (pBEC) were more consistent with previously reported means of 140 Ω × cm^2^ (mBEC) and 449 Ω × cm^2^ (pBEC), respectively [46], although high variability was also observed across pBECs (CV >50%). However, TEER measurements have been described as difficult to translate between studies as they are dependent on factors such as measuring equipment, culture media composition, and temperature [60]. Additionally, Thomsen et al. [46], demonstrated that TEER values do not always correlate with the permeability of the integrity marker mannitol, further complicating the use of TEER as an exclusive indicator of barrier integrity. Despite variable and sometimes lower than previously reported TEER, reflecting low barrier tightness, the transport data clearly showed the presence of an active uptake mechanism across the primary BECs, and observed directional differences (A-to-B >B-to-A) are unlikely to arise from potential paracellular leak, which would affect both directions equally.

A recent meta-analysis of in vitro BBB models describes that BBB modeling is complex, and many transcellular transport models likely have unexamined factors that affect their accuracy in replicating the BBB [61]. For instance, while static BEC monoculture transcellular models are simple and cost-effective, dynamic microfluidics systems and more advanced transcellular models more accurately replicate the in vivo BBB properties. Our study presents evidence of uptake into the human hCMEC/D3 cell line at 200 nM, which is a clinically relevant concentration. The uptake into BBB cell lines has earlier been studied, yet at higher µM concentrations [3, 4, 39, 62]. In hCMEC/D3, the extent of oxycodone uptake was found to be time and concentration-independent in the ranges 2–60 min and 10–5000 nM. In addition, the in vivo microdialysis pilot study, where the transport across the endothelial cells includes release into brain ISF, confirmed concentration-independence of the uptake into endothelial cells, by showing minimal variations in K_p,uu,brain_ across the wide range of unbound plasma concentrations of 95–8022 nM.

The rate of oxycodone uptake into hCMEC/D3 was dependent on both time and concentration, as previously described [3, 4]. The estimated K_m_ of ~ 23 µM obtained in this study was much higher than the oxycodone concentrations used, hence, the value may be uncertain due to the extrapolation. Previous studies have reported K_m_ values for oxycodone uptake in hCMEC/D3 in the range of 89–130 µM [3, 4]. These K_m_ values are much higher than clinically relevant oxycodone plasma concentrations, indicating that the uptake mechanism(s) have high capacity with low risk of saturation and/or drug interactions at clinically relevant concentrations.

We used the hCMEC/D3 model to study the impact of the inflammatory cytokine IL-6 on oxycodone transport into endothelial cells. Our investigation revealed that IL-6 slightly increased the extent of oxycodone uptake, contrary to a significant decrease in oxycodone BBB uptake observed during LPS-induced inflammation in vivo in rats [33]. The 60-minute IL-6 incubation time used in the present study is known to be sufficient to induce changes in BBB functionality in vitro. Earlier in vitro studies have provided information that a 20-minute cytokine treatment, including IL-6, was enough to activate immune responses assessed by activation of Janus Tyrosine Kinase/Signal Transducers and Activators of Transcription (JAK/STAT) signaling pathways in hCMEC/D3 [63]. A meta-analysis showed that various disease BBB models (including BBB cell models treated with amyloid precursor proteins, α-synuclein fibrils, lipopolysaccharide or cytokines, or cultured with glioblastoma cells, or primary BECs from Parkinson disease rat brains) overall exhibited approximately 2-fold higher permeability than corresponding healthy models [61]. However, in hCMEC/D3 studies, only drug uptake via passive and active transport across the cellular membrane is considered. Cytokine treatment has previously been shown to decrease expression levels and functional activity of efflux transporters [64–66], while there are also reports on the increased expression levels and functional activity of efflux transporters [67]. IL-6 is generally not administered in vivo, but LPS is commonly used in research animals to initiate an inflammatory response, of which IL-6 is a main player [33, 35]. Under oxidative stress, using H_2_O_2_-exposed hCMEC/D3, P-gp efflux activity has been shown to decrease due to rapid internalization of the transporter [68]. The increased uptake observed of oxycodone in the present study may therefore also be caused by internalization of P-gp, as oxycodone may interact with P-gp. Discrepancies between in vivo and in vitro data can be explained by the complex interplay of multiple factors, including various pro-inflammatory mediators and physiological changes in vivo, that are not replicated in vitro. This conclusion was also made by Kawase et al. [32], who studied another antiporter substrate diphenhydramine [69]. Overall, IVIV discrepancies may depend on the experimental models used (LPS vs. cytokine treatment) as well as the selected model drugs.

In our study, we did not observe any IL-6 concentration-dependent effects on intracellular uptake of oxycodone into hCMEC/D3. In contrast, transport studies using primary rBECs have shown IL-6–induced barrier disruption with concentration-dependent decreases in TEER [50], and in hCMEC/D3 cells, IL-6 has been reported to downregulate barrier integrity-related genes, including cell junctions, with minor effects on the decrease of ABC transporters’ mRNA expression levels and their efflux activities [70]. A contributing cause of the lack of effect in our study may be the absence of other cell types needed to mediate responses occurring in vivo. Overall, these findings suggest that IL-6 may primarily influence barrier integrity rather than transport activity, and highlight the need for more complex models to define the specific contributions of various cytokines and immune responses. They also emphasize the necessity of in vivo studies to understand the impact of complex pathological conditions on drug transport to the brain in a broader biological context.

Organic cations, including oxycodone and pyrilamine, distribute across membranes via passive diffusion driven by pH partitioning and active uptake via the H^+^/OC antiporter, and as described above, possibly counteracted by efflux transporters, all operating in parallel. To distinguish between molecular transport processes and standardize evaluation across cell types and methods, we have used K_p,uu,cell_ to provide a unified measure for comparison. The comparison of the intracellular accumulation of oxycodone, including binding to cellular components, demonstrated higher accumulation in hCMEC/D3 than in rat brain parenchymal cells with a K_p,u,cell_ of 16.6 and a V_u, brain_ of 3.6 mL/g brain, respectively. However, the extent of uptake at the cellular barriers after compensation for binding was around 1.8 in both cases, confirming that the observed differences in total cell-to-unbound buffer concentration ratios are governed by oxycodone binding properties in brain endothelial and parenchymal cells. These results suggest that while the intracellular accumulation, including binding capacity, may differ, the intracellular accumulation of unbound drug is present to a similar extent in both cell types. Yet, the BBB transport and the intra-brain parenchymal cell uptake are two independent processes and may be governed by different factors.

The estimation of K_p,uu,cell_ in parenchymal and endothelial cells is directly influenced by the assessment of the extent of drug binding in brain tissue and hCMEC/D3 cells. While a dilution factor of 10, commonly used in f_u, brain_ determination, is validated and extensively studied [71], the larger dilution factor of 211.5 used in the determination of hCMEC/D3 f_u, cell_ is associated with higher uncertainty. This factor was derived from microscopic estimates of the cell height and the well surface area, raising concerns about its accuracy. Previously, the dilution factor for estimating drug f_u, cell_ in various cell lines was determined using total protein content in cell lysates and a cell volume of 6.5 µL/mg protein measured as ^14^C-urea space in HEK-293 cells [49, 72, 73]. To our knowledge, no such measurement of cell volume has been reported for hCMEC/D3 cells. If the dilution factor for hCMEC/D3 cells is overestimated, it would lead to an underestimation of f_u, cell_, and consequently an underestimation of K_p,uu,cell_. Despite probable underestimation of the fraction of unbound drug in the cells, K_p,uu,cell_ would still reflect a net active uptake into the cell.

As oxycodone is a basic compound with a pKa of 9.1 [74], and hence subject to lysosomal trapping, it is complicated to distinguish whether the main contributing factor to the intracellular accumulation is due to pH partitioning and/or active uptake at the cell membrane. Data obtained by the brain slice assay demonstrated that the intracellular pH modulators monensin and bafilomycin A1 significantly decreased both oxycodone and pyrilamine uptake into the brain parenchymal cells (K_p,uu,cell_) [75], indicating that lysosomal trapping plays a crucial role for the intracellular disposition of these drugs. Yet, the interplay between lysosomal trapping and active transport across both the cellular as well as lysosomal membranes remains complex, as two transporters, H^+^/OC antiporter and SLC49A4, were recently suggested to be responsible for pyrilamine lysosomal uptake and efflux, respectively [76]. This complexity is further elevated by the fact that the H^+^/OC antiporter-mediated transport, possibly present at cellular and subcellular membranes, is also pH-dependent [1]. Hence, pH modulation may affect not only pH partitioning but also antiporter-mediated uptake.

Additionally, comparing predicted K_p,uu,cell_ values, obtained by the pH partitioning model [74], with experimentally obtained values may provide insights into the contributions of active uptake at the cellular barrier and lysosomal trapping, as the pH partitioning model assumes only passive transport [74]. If the experimentally measured value is above unity and the predicted value is equal to unity or less, the main reason for the intracellular uptake is active uptake, with pH partitioning playing a minor role. The previously predicted K_p,uu,cell_ of 2.9 in brain parenchymal cells is above unity, which indicates that pH partitioning is an important contributor to the intracellular accumulation within the brain [74]. A pH partitioning model is currently not available for brain endothelial cells, but would be helpful for the evaluation of the driving factors for oxycodone uptake into hCMEC/D3.

Based on the experimental findings and predicted partitioning, it is difficult to distinguish if the in vitro uptake observed for oxycodone into endothelial as well as parenchymal cells is governed by active transport mediated by the H^+^/OC antiporter and/or other transporters, or governed by pH partitioning only. From in vivo studies, it is clear that oxycodone is actively transported into the brain across the BBB with K_p,uu,brain_ values above unity, i.e., 2.5 in pigs [13], and 3–4.4 in rats [10, 12], governed by the primary uptake mechanism possibly via the antiporter system [3, 6, 39, 62]. The remaining question is if the antiporter is present at the isolated primary BEC and hCMEC/D3 cell membranes, as earlier suggested in rBECs by Kawase et al. [32] and for hCMEC/D3 by Shimomura et al. [6], and if it is present in the brain parenchymal cells, which has not been proven.

## Conclusion

Our study provides insights into the mechanisms governing the brain distribution of oxycodone, particularly emphasizing the contribution of active transport mechanisms at multiple barriers. Our findings demonstrate that the investigated in vitro BBB models (hCMEC/D3, pBECs, rBECs, mBECs) are reliable tools for assessing substrate uptake in drug development, particularly for H^+^/OC antiporter substrates. The consistency between our in vitro and in vivo data further supports the translational relevance of these models for CNS drug delivery. Importantly, characterizing not only the rate but also the extent of drug cellular transport, with the parameter K_p,uu,cell_ being particularly important in this context, is critical for understanding drug disposition. Overall, this study advances translational aspects of drug delivery to the brain and strengthens the utility of in vitro screening for future CNS drug development and research.

## Supplementary Information

Below is the link to the electronic supplementary material.

Supplementary Material 1

## Data Availability

The datasets used and/or analyzed during the current study are available from the corresponding authors (Irena Loryan and Birger Brodin) on reasonable request.

## Abbreviations

A: Drug amount
A: Compartment Apical compartment
aECF: Artificial extracellular fluid
ANOVA: Analysis of variance
B: compartment Basolateral compartment
BBB: Blood-brain barrier
BEC: Brain endothelial cell
BSA: Bovine serum albumin
C: Concentration
CNS: Central nervous system
CV: Coefficient of variation
ΔC: Concentration gradient
DMEM: Dulbecco’s Modified Eagle’s Medium
EC: Endothelial cell
ER: Efflux ratio
FBS: Fetal bovine serum
f_u_: Fraction of unbound drug
f_u,brain_: Fraction of unbound drug in brain
f_u,cell_: Fraction of unbound drug in the cell
H^+^/OC: Antiporter Putative-proton coupled organic cation antiporter
HBSS: Hanks′ Balanced Salt solution
hCMEC/D3: Immortalized human brain capillary endothelial cell line
IVIV: In vitro – in vivo
IS: Internal standard
ISF: Interstitial fluid
J: Flux
J_max_: Maximum flux
K_m_: Michaelis-Menten constant
K_p,u,cell_: Total intracellular-to-unbound extracellular concentration ratio
K_p,uu,brain_: Unbound brain ISF-to-plasma concentration ratio
K_p,uu,cell_: Unbound intra-to-extracellular concentration ratio
K_p,uu,lyso_: Unbound lysosome-to-cytosol concentration ratio
LLOQ: Lower limit of quantification
mBEC: Primary mouse brain endothelial cell
P: Permeability
pBEC: Primary pig brain endothelial cell
PBS: Phosphate-buffered saline
PC: Parenchymal cell
PDS: Sterile Plasma Derived Bovine Serum
pen/strep: Penicillin/streptomycin
QC: Quality control
rBEC: Primary rat brain endothelial cell
SA: Surface area
SD: Standard deviation
t: Sampling time
TEER: Transendothelial electrical resistance
TR-BBB13: Immortalized rat brain capillary endothelial cell line
UPLC-MS/MS: Ultraperformance liquid chromatography-tandem mass spectrometry
V: Volume
V_u,brain_: Unbound volume of distribution in the brain
