# Supplementary material for: Comparative analysis of the uptake of the H+/OC antiporter substrate oxycodone across and into brain endothelial and parenchymal cells with in vitro–in vivo extrapolation
Source: Fluids Barriers CNS. 2025 Oct 30;22:109. doi: 10.1186/s12987-025-00726-w (PMC12577036; doi:10.1186/s12987-025-00726-w)
Supplement: Supplementary file 1 — Supplementary Material 1 [file 12987_2025_726_MOESM1_ESM.pdf]

Comparative analysis of the uptake of the H^+/OC antiporter substrate oxycodone across and into brain endothelial and parenchymal cells with *in vitro–in vivo* extrapolation

Frida Bällgren[1,2], Nana Svane[3], Aghavni Ginosyan[1], Alberte Bay Villekjær Pedersen[3], Shannuo Li[1,4], Jessica Mahajan[1,5], Morten Schallburg Nielsen[6], Birger Brodin[*3] and Irena Loryan[*1]

[*]Shared last authorship

[1]Translational Pharmacokinetics/Pharmacodynamics group (tPKPD), Department of Pharmacy, Uppsala University, Husargatan 3, 752 37 Uppsala, Sweden

[2]Current affiliation: Department of Pharmacy, Science for Life Laboratory Drug Discovery and Development (SciLifeLab DDD), Uppsala University, Uppsala Sweden

[3]CNS Drug Delivery and Barrier Modelling group (CNSBM), Department of Pharmacy, University of Copenhagen, Copenhagen, Denmark

[4]Current affiliation: Department of Molecular Pharmaceutics, College of Pharmacy, University of Utah, Salt Lake City, UT, 84112, USA

[5]Current affiliation: School of Applied Sciences, Abertay University, Bell Street, Dundee, DD1 1HG, Scotland, United Kingdom

[6]Department of Biomedicine, Aarhus University, Aarhus, Denmark

Corresponding authors:

Irena Loryan and Birger Brodin

Contents

1. Supplementary Materials: *In vivo* microdialysis for assessment of oxycodone concentration impact on the extent of uptake across the BBB

2. Supplementary Materials: Tables S1-S5

3. Supplementary materials: Figures and figure legends, Figures S1-S3

4. References

Supplementary Material

1. *In vivo* microdialysis for assessment of oxycodone concentration impact on the extent of uptake across the BBB

A rat microdialysis pilot study (N=1) was performed to evaluate extent of oxycodone BBB transport, assessed by $K_{p,uu,brain}$, at three different oxycodone dose levels administered intravenously (i.v.). The microdialysis experiment including preparations were performed as previously described (1). After a one-week acclimatization period, microdialysis surgery was performed and 24 hours later, the experiment was initiated.

1.1. Microdialysis surgery

At the time of surgery, the rat was anesthetized with 5 % isoflurane, and the body temperature was maintained at 37°C. The left femoral vein was catheterized (polyethylene (PE)-50 tubing fused with silastic tubing) for oxycodone administration, and the left femoral artery was catheterized (PE-50 tubing fused with PE-10 tubing) for blood sampling. A 10 mm CMA 20 Elite probe was implanted in the right jugular vein and secured with sutures to the pectoral muscles (CMA Microdialysis AB, Kista, Sweden). To place the brain probe, the rat was placed in a stereotaxic instrument, and the skull was exposed. A 3 mm CMA 12 Elite probe was inserted into the right striatum via a guide cannula using the coordinates +0.8 mm anteroposterior and −2.7 mm lateral to bregma, and −3.8 mm dorsoventral to the surface of the brain (CMA Microdialysis AB, Kista, Sweden). The probe was fixed to the skull with a screw and dental cement. Following surgery, the rat was placed in a CMA/120 system for freely moving animals (CMA Microdialysis AB, Kista, Sweden) to recover for 24 hours with free access to food and water.

1.2. Microdialysis experiment and dosing regimens

The microdialysis experiment was conducted 24 hours after surgery, and was divided into six time periods (Fig. S3), i) a 60-minute stabilization period, ii) a 60-minute low-dose steady-state period (0.048 mg/kg loading dose, 0.108 mg/kg/h maintenance dose), iii) a 60-minute washout period, iv) a 60-minute intermediate-dose steady-state period (0.24 mg/kg loading dose, 0.539 mg/kg/h maintenance dose), v) a 120-minute washout period, and (vi) a 60-minute high-dose steady-state period (1.2 mg/kg loading dose, 2.7 mg/kg/h maintenance dose). Throughout the experiment, the brain and blood probes were perfused with CNS Ringer solution (145 mM NaCl, 0.6 mM KCl, 1 mM MgCl2·6H2O, 1.2 mM CaCl2, 0.2 mM ascorbic acid, 2 mM KH2PO4 and K2HPO4, pH 7.4) spiked with 44 ng/mL of the calibrator oxycodone-D3, at a flow rate of 1 µL/min using a CMA 400 Syringe Pump (CMA Microdialysis AB, Kista, Sweden). Retrodialysis by calibrator was used to determine the recoveries across the probe membranes (2). For all dose levels, oxycodone was administered as an i.v. infusion loading dose over two minutes, followed by a maintenance dose over the remaining 60 minutes. The lowest dose aimed to achieve the lowest quantifiable by our UPLC-MS/MS method blood concentrations, while the highest dose aimed to reach the highest concentrations without causing toxicity, maintaining a twenty-five-fold range in $C_{ss,plasma}$ between the lowest and the highest doses. The infusion was delivered using a Harvard 22 pump (Harvard Apparatus Inc., Holliston, MA) with the infusion rates calculated based on the targeted $C_{ss,plasma}$ and previously reported pharmacokinetics (1).

Dialysate, i.e., probe perfusate, samples from both brain and blood probes were collected in pre-weighed polypropylene microvials with polyurethane caps at 10-minute intervals throughout the experiment (AgnTho's, Lidingö, Sweden). After collection, each sample was immediately weighed, capped, and stored at 6°C until bioanalysis the next day. Blood samples from the femoral artery were collected before the stabilization period and at 5, 35, 55, 125, 155, 175, 305, 335, and 355 minutes after the start of the infusion, using pre-heparinized Eppendorf tubes (5 µL 5000 IU/mL heparin; Eppendorf, Hamburg, Germany). These blood samples were immediately centrifuged at

10,000 rpm for 5 minutes. Plasma was transferred to non-heparinized 1.5 mL polypropylene Eppendorf tubes and stored at -20°C pending bioanalysis.

Terminal blood samples were collected from the heart using a heparinized Vacutainer (6 mL, Polyethylene terephthalate, Vacutest, Kima; Azergrande (PD), Italy). After terminal blood sampling, the brain was isolated, visually examined for correct probe placement, cleaned from meninges and blood vessels, and stored at -80°C pending bioanalysis.

1.3. Microdialysis quantification and data analysis

Dialysate and plasma samples containing oxycodone and oxycodone-D3 (utilized solely in dialysate samples) were quantified using ultraperformance liquid chromatography-tandem mass spectrometry (UPLC-MS/MS) according to the previous method as described in Materials and Methods (1). For samples collected during the microdialysis experiment, calibration curves were constructed in Ringer solution and plasma, respectively, at concentrations of 0.5-1000 ng/mL. The multiple reaction monitoring (MRM) transition monitored for oxycodone-D3 was 319.11 → 301.1 m/z.

To obtain unbound concentrations (C_u) from the *in vivo* microdialysis experiment, the dialysate concentrations were converted to C_u in the brain and blood, respectively, by adjusting for the drug recovery across the probe membrane. The recovery was monitored by retrodialysis by calibrator (oxycodone-D3) throughout the experiment, and due to molecular similarities, the recovery of oxycodone was assumed to be the same as that for the calibrator. The recovery of the calibrator was calculated as:

$$Recovery = {(C_{in} - C_{out})}/{C_{in}} \qquad \text{(Eq. S1)}$$

Where C_{in} is the mean calibrator concentration in the perfusion solution entering the probe, sampled from the probe perfusion syringes before and after the experiment. C_{out} is the mean calibrator concentration in the dialysate samples exiting the brain and blood probes, collected from each probe throughout the experiment. Mean recoveries were calculated for the brain probe (3 mm)

and the blood probe (10 mm) to 6.7 and 50%, respectively, and further used to obtain the C_u in each dialysate sample:

$$C_u = {C_{dialysate}}/{Recovery} \qquad \text{(Eq. S2)}$$

The unbound partition coefficient $K_{p,uu,brain}$ was based on the unbound concentrations obtained during steady-state, in brain interstitial fluid (ISF, $C_{u,brain,ss}$) and blood ($C_{u,blood,ss}$):

$$In\ vivo\ K_{p,uu,brain} = {C_{u,brain,ss}}/{C_{u,blood,ss}} \qquad \text{(Eq. S3)}$$

2. Supplementary Materials: Tables

Table S1. Transendothelial electrical resistance (TEER, Ω x cm^2) across the primary porcine brain endothelial monolayer measured before and after the experiment for each condition (before and after) and directions.

**TEER (Ω x cm^2)**	**Oxy 200 nM (A→B)**		**Oxy 200 nM + pyr 200 µM (A→B)**		**Oxy 200 nM (B→A)**	
	Before	*After*	*Before*	*After*	*Before*	*After*
**B1R1**	532.6	544.3	NA	NA	659.2	663.3
**B1R2**	864.2	885.0	NA	NA	719.7	723.7
**B1R3**	316.8	327.9	NA	NA	763.4	743.9
**B2R1**	224.8	267.1	235.5	256.0	218.4	211.0
**B2R2**	228.5	225.5	430.1	453.6	195.2	224.8
**B2R3**	176.7	189.5	228.5	227.5	135.4	111.6
**B3R1**	304.4	312.5	407.9	353.5	318.5	305.4
**B3R2**	316.8	290.0	170.7	150.2	312.8	273.5
**B3R3**	439.5	388.1	254.4	222.1	365.2	323.6

B=batch, R=replicate, NA=not available. These are adjusted by blank TEER (blank filter ranged between 313-342 Ω, corresponding to ~110 Ω×cm^2) subtraction, and multiplied by the filter area of the cell culture support (0.336 cm^2). TEER was measured before and after the experiment for each condition, i.e, cells incubated with oxycodone (oxy) and pyrilamine (pyr), in both apical-to-basolateral (A→B) and basolateral-to-apical (B→A) directions. On average, TEER was 98±9% after the experiment, compared to before.

Table S2. Transendothelial electrical resistance (TEER, Ω x cm^2) across the primary rat brain endothelial monolayer measured before and after the experiment for each condition (before and after) and directions.

TEER (Ω x cm^2)	Oxy 200 nM (A→B)		Oxy 200 nM + pyr 200 µM (A→B)		Oxy 200 nM (B→A)		^3H-pyr 1 µCi/mL (A→B)	^3H-pyr 1 µCi/mL + pyr 200 µM (A→B)
	Before	*After*	*Before*	*After*	*Before*	*After*	*Before*	*Before*
**B1R1**	79.5	86.4	48.6	36.3	76.4	64.2	52.9	54.9
**B1R2**	73.4	90.7	82.5	72.9	101.3	114.6	54.6	61.3
**B1R3**	82.5	67.2	64.3	41.0	-0.8*	-2.0*	51.6	41.5
**B1R4**	NA	NA	NA	NA	-0.8*	-2.0*	NA	NA
**B2R1**	15.0	21.2	26.0	33.0	NA	NA	22.3	26.7
**B2R2**	25.4	45.4	27.4	47.7	NA	NA	51.9	25.0
**B2R3**	22.7	30.6	37.1	19.2	NA	NA	27.7	35.1
**B3R1**	21.0	12.8	24.4	11.4	NA	NA	15.3	35.1
**B3R2**	10.2	15.5	35.1	6.0	NA	NA	21.7	15.0
**B3R3**	20.0	12.8	18.0	40.0	NA	NA	26.0	24.7

B=batch, R=replicate, NA=not available. These are adjusted by blank TEER (blank filter ranged between 204-287 Ω, corresponding to ~83 Ω×cm^2) subtraction, and multiplied by the filter area of the cell culture support (0.336 cm^2). TEER was measured before and after the experiment for each condition, i.e, cells incubated with oxycodone (oxy) and pyrilamine (pyr), in both apical-to-basolateral (A→B) and basolateral-to-apical (B→A) directions, except when radioactivity was used, in which only a measurement before the experiment was performed. On average, TEER was 129±61% after the experiment, compared to before. *Despite negative TEER measurements in two wells, the data from these wells were included in the final analysis as the data were consistent with those from other wells under the same experimental conditions.

Table S3. Transendothelial electrical resistance (TEER) across the primary mouse brain endothelial monolayer measured before and after the experiment for each condition (before and after) and directions.

**TEER (Ω x cm^2)**	**Oxycodone 200 nM (A→B)**		**Oxycodone 200 nM + pyrilamine 200 µM (A→B)**	
	Before	*After*	*Before*	*After*
**B1R1**	101.5	103.5	150.9	102.6
**B1R2**	153.9	119.8	95.9	92.6
**B1R3**	132.0	NA	98.8	93.6

B=batch, R=replicate, NA=not available. These are adjusted by blank TEER (blank filter ranged between 434-510 Ω, corresponding to ~161 Ω×cm^2) subtraction, and multiplied by the filter area of the cell culture support (0.336 cm^2). On average, TEER was 88±14% after the experiment, compared to before.

Table S4. Statistical test details on comparison of $K_{p,u,cell}$ values of oxycodone in hCMEC/D3 cells with and without interleukin-6 (IL-6) exposure.

**Ordinary one-way ANOVA with Tukey's multiple comparisons test**						
F	10.8					
P value	0.0018					
P value summary	**					
Significant diff. among means (P < 0.05)?	Yes					
R squared	0.7642					
**Data summary**						
Number of treatments (columns)	4					
Number of values (total)	14					
Number of families	1					
Number of comparisons per family	6					
	**IL6 1 ng/mL vs IL6 10 ng/mL**	**IL6 1 ng/mL vs IL6 100 ng/mL**	**IL6 10 ng/mL vs IL6 100 ng/mL**	**IL6 1 ng/mL vs Control**	**IL6 10 ng/mL vs Control**	**IL6 100 ng/mL vs Control**
**Tukey's multiple comparisons test**						
Mean Diff,	0.2657	1.685	1.419	8.051	7.786	6.366
95,00% CI of diff,	-5,637 to 6,169	-4,218 to 7,588	-4,484 to 7,322	2,771 to 13,33	2,506 to 13,07	1,087 to 11,65
Below threshold?	No	No	No	Yes	Yes	Yes
Summary	ns	ns	ns	**	**	*
Adjusted P Value	0.999	0.8185	0.8807	0.0041	0.0052	0.0184
**Test details**						
Mean 1	23.42	23.42	23.16	23.42	23.16	21.74
Mean 2	23.16	21.74	21.74	15.37	15.37	15.37
Mean Diff,	0.2657	1.685	1.419	8.051	7.786	6.366
SE of diff,	1.93	1.93	1.93	1.726	1.726	1.726
n1	3	3	3	3	3	3
n2	3	3	3	5	5	5
q	0.1947	1.235	1.04	6.598	6.38	5.217
DF	10	10	10	10	10	10

Table S5. Oxycodone concentration-independent intra-brain distribution. Data are obtained using rat brain slice assay at different starting incubation concentrations (minimum 3 rat brains per concentration).

Oxycodone concentration (nM)	$V_{u,brain}$ (mL/g brain)
100	3.9±0.3
500	4.2±0.2
1000	3.9±0.1
5000	3.8±0.2

3. Supplementary materials: Figures and figure legends

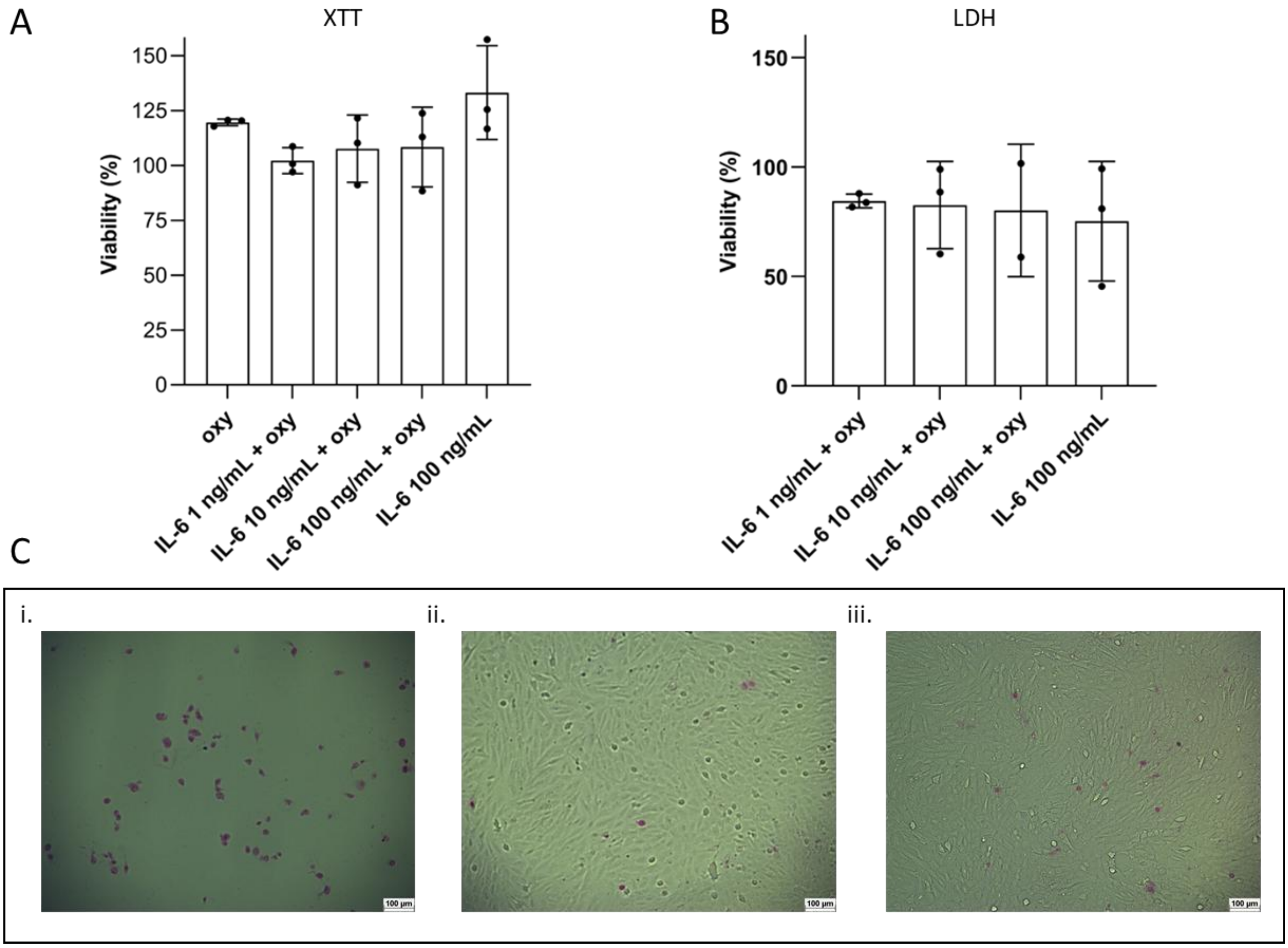

Figure S1. Viability of hCMEC/D3 cells pre-treated with interleukin-6 (IL-6, 60 minutes) and/or oxycodone (oxy, 200 nM, 5 minutes). A. Methoxynitrosulfophenyl-tetrazolium carboxanilide (XTT) assay. **B.** Lactate dehydrogenase (LDH) assay. **C.** Representative images of erythrosine B staining of hCMEC/D3: i. Dead cell control, ii. Live cell control, iii. 60 minutes IL-6 (100 ng/mL) pre-treatment with 5-min oxycodone incubation at 200 nM. Repeated measures one-way ANOVA with the Geisser-Greenhouse correction and Dunnett's multiple comparisons test was used to compare the viability of hCMEC/D3 between control and IL-6 pre-treatments obtained by the respective assay (XTT and LDH). See details on statistical analysis in Table S4.

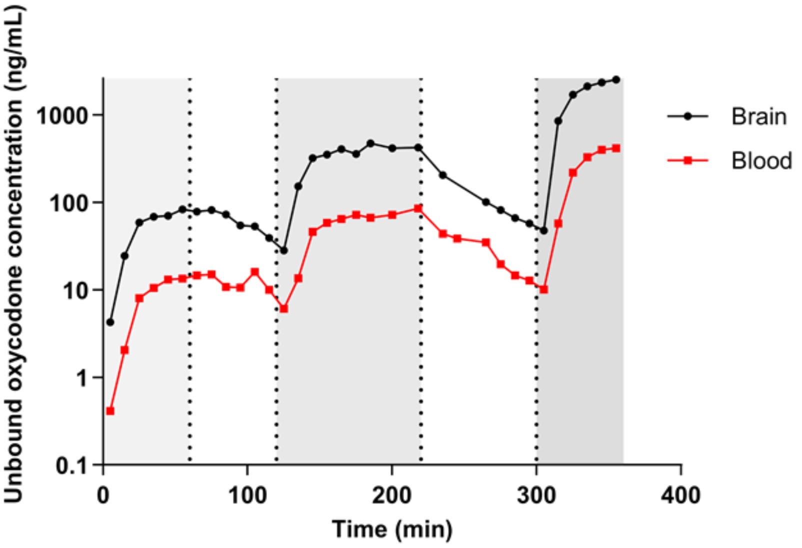

Figure S2. Unbound oxycodone blood and brain concentrations over time. Concentrations obtained from the microdialysis exploratory study by administering three different oxycodone doses of 0.2 mg/kg (0-60 minutes), 1.1 mg/kg (120-220 minutes) and 3.9 mg/kg (300-360 minutes) (N=1).

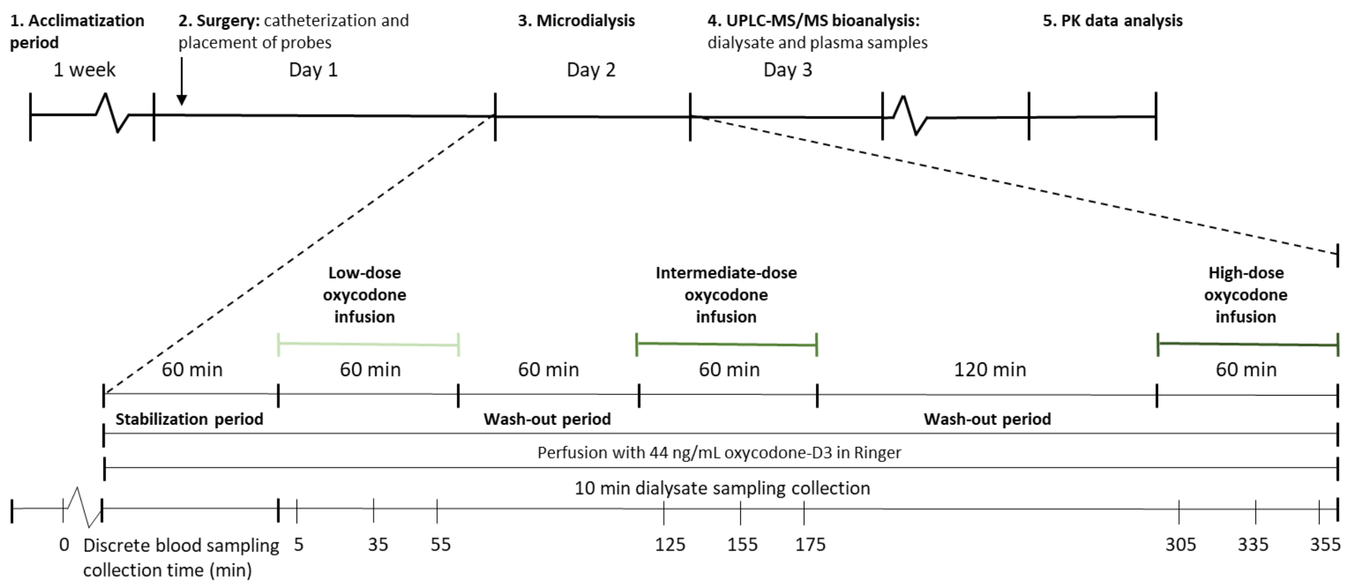

Figure S3. **Schematic overview of the microdialysis pilot study assessing the extent of oxycodone BBB transport.** The rat was first acclimatized for one week. On day 1, the microdialysis surgery was performed. On day 2, the microdialysis experiment was performed. The microdialysis experiment aimed to evaluate oxycodone uptake at three different doses administered as intravenous infusions (Low-dose: 0.048 mg/kg loading dose, 0.108 mg/kg/h maintenance dose; Intermediate-dose: 0.24 mg/kg loading dose, 0.539 mg/kg/h maintenance dose; High-dose: 1.2 mg/kg loading dose, 2.7 mg/kg/h maintenance dose). The probes were perfused with 44 ng/mL oxycodone-D3 in Ringer solution. Dialysate, i.e., probe perfusate, were collected in 10-minute intervals, and plasma was collected by blood sampling at specified time points. Oxycodone and oxycodone-D3 were quantified in the samples using UPLC-MS/MS.

4. References

1. Bällgren F, Hammarlund-Udenaes M, Loryan I. Active Uptake of Oxycodone at Both the Blood-Cerebrospinal Fluid Barrier and The Blood-Brain Barrier without Sex Differences: A Rat Microdialysis Study. Pharmaceutical research. 2023;40(11):2715-30.DOI: 10.1007/s11095-023-03583-0.

2. Bouw MR, Hammarlund-Udenaes M. Methodological aspects of the use of a calibrator in in vivo microdialysis-further development of the retrodialysis method. Pharmaceutical research. 1998;15(11):1673-9.DOI: 10.1023/a:1011992125204.
